# Phase-engineering the Andreev band structure of a three-terminal Josephson junction

**DOI:** 10.1038/s41467-023-42356-6

**Published:** 2023-10-25

**Authors:** Marco Coraiola, Daniel Z. Haxell, Deividas Sabonis, Hannes Weisbrich, Aleksandr E. Svetogorov, Manuel Hinderling, Sofieke C. ten Kate, Erik Cheah, Filip Krizek, Rüdiger Schott, Werner Wegscheider, Juan Carlos Cuevas, Wolfgang Belzig, Fabrizio Nichele

**Affiliations:** 1 IBM Research Europe—Zurich, 8803 Rüschlikon, Switzerland; 2 Fachbereich Physik, Universität Konstanz, D-78457 Konstanz, Germany; 3 Laboratory for Solid State Physics, ETH Zürich, 8093 Zürich, Switzerland; 4 Departamento de Física Teórica de la Materia Condensada and Condensed Matter Physics Center (IFIMAC), Universidad Autónoma de Madrid, E-28049 Madrid, Spain; 5 Present Address: Institute of Physics, Czech Academy of Sciences, 162 00 Prague, Czech Republic

**Keywords:** Superconducting properties and materials, Superconducting devices

## Abstract

In hybrid Josephson junctions with three or more superconducting terminals coupled to a semiconducting region, Andreev bound states may form unconventional energy band structures, or Andreev matter, which are engineered by controlling superconducting phase differences. Here we report tunnelling spectroscopy measurements of three-terminal Josephson junctions realised in an InAs/Al heterostructure. The three terminals are connected to form two loops, enabling independent control over two phase differences and access to a synthetic Andreev band structure in the two-dimensional phase space. Our results demonstrate a phase-controlled Andreev molecule, originating from two discrete Andreev levels that spatially overlap and hybridise. Signatures of hybridisation are observed in the form of avoided crossings in the spectrum and band structure anisotropies in the phase space, all explained by a numerical model. Future extensions of this work could focus on addressing spin-resolved energy levels, ground state fermion parity transitions and Weyl bands in multiterminal geometries.

## Abstract (web summary)

The authors study Andreev bound states (ABSs) in 3-terminal InAs/Al Josephson-junction devices. They find signatures of hybridization between two ABSs, with band structure tunable by electric currents that generate magnetic fluxes threading superconducting loops in the device.

## Introduction

In a normal conductor interfacing two or more superconductors, charge carriers at energies within the superconducting gap are confined by Andreev reflection processes occurring at the interfaces^1^. As a result, resonant sub-gap electronic excitations known as Andreev bound states (ABSs) arise in the normal region, enabling transport of a Josephson supercurrent between the superconducting terminals^2,3^. These discrete ABS levels were proposed as a basis for quantum computing applications^4–7^. More recently, ABSs have been the subject of intense experimental investigation in various material platforms^8–15^, culminating in the coherent control of Andreev pair^16,17^ and spin^18,19^ qubits. While these studies focused on Josephson junctions (JJs) with two superconducting terminals, where the ABS energies depend on a single superconducting phase difference, multiterminal JJs (MTJJs) have also emerged as a promising alternative. In the presence of *N*≥3 terminals, the ABS band structure spanned by the *N*−1 independent phase differences was predicted to exhibit a plethora of phenomena, including lifting of the spin degeneracy^20^, ground state fermion parity transitions^20^, Weyl singularities^21–25^ and other topological properties^26–31^. Moreover, MTJJs were proposed to realise Andreev molecules^32–35^—a system where single ABSs overlap and form hybridised energy levels—and explored as a platform to generate Cooper quartets^36–40^. Extensive experimental work was also conducted on MTJJs in the presence of DC current bias^41–44^ and microwave irradiation^45^. While tunnelling spectroscopy of metallic three-terminal JJs (3TJJs) was performed previously^46^, establishing phase control over the superconducting proximity gap, further investigation is needed to understand the properties of ABS bands in multiterminal devices. The perspective of engineering synthetic band structures by exploiting the higher dimensionality of the phase space is particularly attractive, as it could enable effects unattainable in two-terminal geometries.

Here, we report on an experimental realisation of superconductor–semiconductor 3TJJs and study ABSs in the system with tunnelling spectroscopy. Owing to the independent control over two superconducting phase differences, we probe the Andreev band structure in the two-dimensional (2D) phase space and find signatures of hybridisation between highly transmissive ABSs, resulting in the formation of an Andreev molecule. Our measurements are supported by a theoretical model and demonstrate the feasibility of Andreev matter and phase-engineering of Andreev bands in hybrid nanostructures.

## Results

### Implementation of phase-controllable 3TJJ

**Fig. 1:**
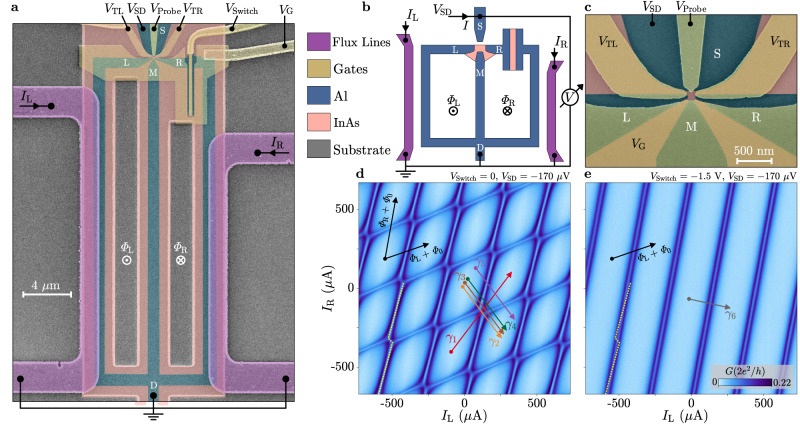
Experimental setup and tunnelling conductance in the two-dimensional phase space. **a** False-coloured scanning electron micrograph of a device identical to that under study, defined by selective removal of the Al (blue), exposing the semiconductor below (pink). Gates (yellow) and flux-bias lines (purple) were deposited on a uniform dielectric layer (not visible). Bias voltage *V*_SD_, gate voltages *V*_*α*_ (*α* ∈ {TL, TR, Probe, Switch, G}), left (right) flux-bias line current *I*_L(R)_ and external magnetic flux threading the left (right) superconducting loop Φ_L(R)_ are indicated. **b** Schematic representation of the device and the measurement setup. **c** Zoom-in of **a** near the three-terminal Josephson junction (3TJJ) region. **d**, **e** Differential conductance *G* between tunnelling probe and 3TJJ measured as a function of the currents *I*_L_ and *I*_R_ injected into the flux-bias lines, at fixed voltage bias *V*_SD_ = − 170 μV. In **d** (**e**), the switch junction is in the ON (OFF) state, *V*_Switch_ = 0 (*V*_Switch_ = − 1.5 V). The directions of the black arrows indicate the periodicity axes, along which the external magnetic fluxes Φ_L_ and Φ_R_ vary independently. Each black arrow represents the addition of one superconducting flux quantum Φ_0_ = *h*/2*e* (where *h* is the Planck constant and *e* the elementary charge) to the corresponding flux. The dotted yellow line follows a Φ_L_-dependent resonance in **d** and is replicated in **e** to highlight the slope difference. The coloured arrows labelled *γ*_1-6_ define the linecuts shown in Fig. 2.

The device under investigation, shown in Fig. 1a–c, was realised in an InAs/Al heterostructure^47,48^. Selective etching of the Al layer defined three superconducting terminals (labelled L, M and R) coupled to a normal region, constituting a hybrid 3TJJ. The three terminals were connected to a common node (D) forming two closed loops; while leads L and M were directly connected to D via Al strips, a superconductor–normal–superconductor (SNS) JJ was integrated on terminal R. The junction, with a length of 40 nm and a width of 5 μm, was designed to have a critical current much larger than that existing between any pairs of L, M and R, hence the superconducting phase difference across the junction is neglected for the following discussion. A fourth superconducting lead (S) was employed as a probe to enable DC tunnelling spectroscopy of the sub-gap states in the 3TJJ. Metallic gate electrodes and flux-bias lines were patterned on top of an insulating layer uniformly deposited across the entire sample. The transmission between the probe and the 3TJJ was tuned via two gates energised by the common voltage ${V}_{{{{{{{{\rm{T}}}}}}}}}\equiv {V}_{{{{{{{{\rm{TL}}}}}}}}}={V}_{{{{{{{{\rm{TR}}}}}}}}}$, which was set to − 1.07 V to enter the tunnelling regime: in this configuration, the probe was weakly coupled to the 3TJJ and its influence on the rest of the circuit was limited. Gate tuning of the SNS junction enabled its operation as a switch, with the ON (OFF) state defined by *V*_Switch_ = 0 (*V*_Switch_ = −1.5 V). This allowed for the connection or disconnection of terminal R from D, hence electrostatically selecting between a three-terminal (switch ON) and a two-terminal (switch OFF) configuration. Two additional gate voltages were kept to *V*_Probe_ = 150 mV and *V*_G_ = 50 mV. A current *I*_L(R)_ injected into the left (right) flux-bias line generated an external magnetic flux Φ_L(R)_ threading the left (right) superconducting loop, thus tuning the phase difference between L (R) and M, and enabling control over the whole 2D phase space. As schematically depicted in Fig. 1b, a DC voltage bias *V*_SD_ was applied to the probe S and the differential tunnelling conductance *G* was measured between S and D with standard lock-in techniques. Experiments were performed in a dilution refrigerator with base temperature below 10 mK. Further details about materials, fabrication and measurements are provided in the “Methods” section.

### Probing the Andreev band structure in the 2D phase space

In Fig. 1d, e, the voltage bias was set to − 170 μV and the tunnelling conductance was measured as a function of the currents *I*_L_ and *I*_R_ injected into the flux-bias lines, resulting in a scan over an extended region of the 2D phase space at constant energy. Resonances in conductance correspond to peaks in the density of states (DOS) of the normal region under study^8,15^ and represent ABSs in the 3TJJ. Each state is intersected twice per period at *V*_SD_ = − 170 μV (i.e., at an energy below its maximum), giving rise to the characteristic appearance of pairs of lines in these maps. When the switch is set to the ON state (Fig. 1d), we observe periodic features as a function of both *I*_L_ and *I*_R_, attributed to the presence of two distinct ABSs whose energies disperse with Φ_L_ and Φ_R_, respectively. The cross-dependence between the flux-bias lines accounts for the finite slope of the (Φ_L_, Φ_R_) axes, as indicated by the black arrows. Remarkably, resonances associated to different ABSs are connected to each other in proximity of the intersections, forming closed diamond-like loops and avoided crossings at the corners of the diamonds. Each state undergoes a phase shift when intersecting the other, defining a zig-zag trend. Next, we set the switch junction voltage to −1.5 V (OFF, Fig. 1e) and observe that the ABS resonances depending on Φ_R_ disappear, while the complex 2D periodic pattern is transformed into a simple structure depending on a single flux. Notably, the slope of the Φ_L_-dependent resonances differs between panels d and e (see dotted yellow lines), which is compatible with the periodic phase shift present only when the switch is ON.

### Andreev dispersion along phase space linecuts

**Fig. 2:**
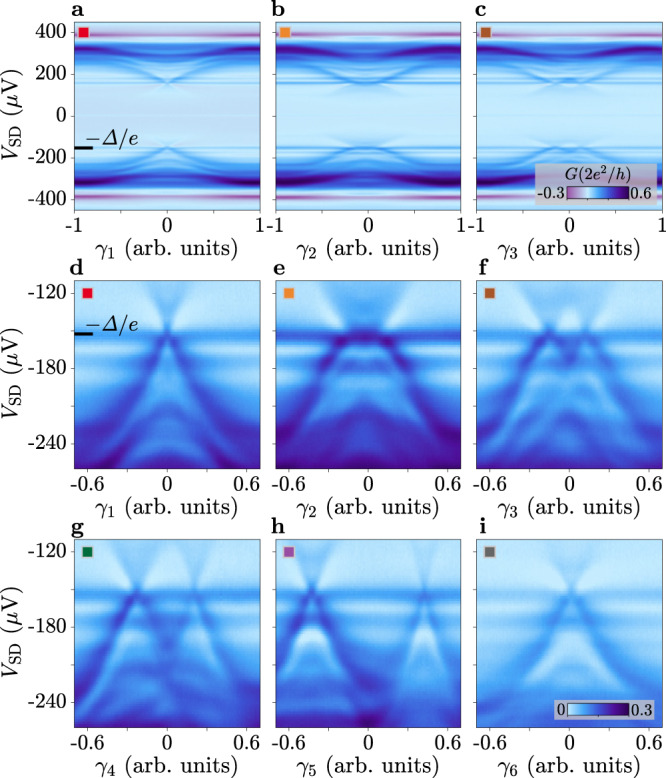
Tunnelling conductance spectra along phase space linecuts. **a**–**c** Differential tunnelling conductance *G* measured as a function of voltage bias *V*_SD_ along the linecuts *γ*_*i*_ (coloured arrows in Fig. 1d), with *V*_Switch_ = 0. The lower edge of the transport gap − Δ/*e*, due to the superconducting tunnelling probe, is indicated by the black marker. **d**–**h**, As **a**–**c**, but plotted over restricted ranges of *V*_SD_ and *γ*_*i*_. **i**, As **d**–**h**, but along linecut *γ*_6_ (defined in Fig. 1e), for *V*_Switch_ = −1.5 V. The colourbar in **i** applies to **d**–**i**.

With the phase space overview acquired at constant voltage bias, we select cut lines *γ*_1-6_ (arrows in Fig. 1d, e) along which the tunnelling conductance is measured as a function of *V*_SD_. The full datasets along *γ*_1_, *γ*_2_ and *γ*_3_ are displayed in Fig. 2a–c. Each shows a transport gap 2Δ/*e* ≈ 310 μV (*e* is the elementary charge), consistent with a superconducting gap of the Al probe Δ ≈ 155 μeV, and an electron–hole-symmetric spectrum revealing phase-dependent ABSs. The presence of regions with finite conductance at *e*∣*V*_SD_∣ ≲ Δ is ascribed to broadened features in the DOS of the superconducting probe for energies ∣*E*∣ ≈ Δ. This could be due to a combination of quasiparticle-lifetime broadening^49^ and additional subgap bound states forming between probe and 3TJJ. On either side of the spectrum, we notice two differential conductance peaks at *V*_SD_ = ±155 μV and ±175μV, whose position in bias does not vary appreciably with *γ*_*i*_. The first is attributed to the multiple Andreev reflection peak at ± Δ/*e*, while the second, specific to this device, might be related to mesoscopic defects in the tunnelling probe or to a region in the device with a larger superconducting gap. These peaks provide a contribution to the measured differential conductance, adding to that of ABS resonances and spectroscopy background. This accounts for the intensity modulation of these peaks depending on *γ*_*i*_. Further supported by measurements at different tunings of the probe (see Supplementary Note 4), we do not observe a distortion of the ABS dispersion when the states cross the peaks. In Fig. 2d–i, all six linecuts are plotted in restricted *V*_SD_ and *γ*_*i*_ ranges for better clarity. In each case, ABSs approach the transport gap edge very closely, which indicates near-unity transmission. We also note that such highly transmissive ABSs intersect *V*_SD_ = −170 μV twice per period, thus accounting for the pairs of resonances in Fig. 1d, e. Interestingly, when comparing the *γ*_1_ and *γ*_2_ linecuts (Fig. 2d, e), we find anisotropic ABS phase dispersion in the vicinity of (*π*, *π*) phase (*γ*_1,2_ = 0), with a narrow, cusp-like shape versus a broader and flatter peak, respectively. In the spectroscopy measurements performed along *γ*_3-5_, i.e., lines parallel to *γ*_2_ but offset from an intersection point of Fig. 1d, two distinct highly transmissive ABSs appear. When their separation in phase is small, the states partially mix and an avoided crossing is observed (Fig. 2f), an effect which is weaker at larger separation (Fig. 2g), until it is completely suppressed (Fig. 2h). Finally, linecut *γ*_6_, where the switch junction is kept in the OFF state, reveals a single highly transmissive ABS (Fig. 2i).

### Andreev molecule model and numerical simulations

**Fig. 3:**
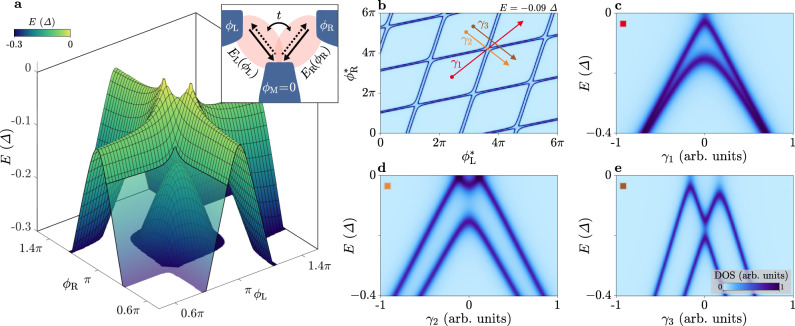
Theoretical model of coupled Andreev bound states and Andreev molecule. **a** Simulated Andreev bound state (ABS) energy bands as a function of the superconducting phase differences *ϕ*_L_ and *ϕ*_R_ for negative energies *E* ≤ 0. The band structure at positive energies (not shown) is specular due to electron–hole symmetry. Inset: simplified schematic of the model. Three superconducting terminals (blue), with phases *ϕ*_L_, *ϕ*_R_ and *ϕ*_M_ = 0, are interconnected via two Andreev channels (pink) that are located between the middle lead and either the left or the right lead. The energy *E*_L(R)_ of the left (right) ABS depends on the phase difference *ϕ*_L(R)_ − *ϕ*_M_ ≡ *ϕ*_L(R)_. The two ABSs are coupled to each other, enabling hybridisation of their energy levels, described by the parameter *t*. A full description of the model is provided in the Supplementary Note 1. **b** Simulated density of states (DOS) at fixed energy *E* = − 0.09Δ as a function of the cross-coupled superconducting phase differences ${\phi }_{{{{{{{{\rm{L}}}}}}}}}^{*}$ and ${\phi }_{{{{{{{{\rm{R}}}}}}}}}^{*}$, defined as linear combinations of *ϕ*_L_ and *ϕ*_R_ to better represent the experimental data (see Supplementary Note 1 for more details). **c**–**e** Simulated DOS as a function of energy along the linecuts of the phase space *γ*_1−3_, defined in **b** (coloured arrows).

To simulate ABSs arising in a 3TJJ, we study a minimal theoretical model comprising three superconducting terminals with phases *ϕ*_L_, *ϕ*_R_ and *ϕ*_M_, as schematically sketched in the inset of Fig. 3a. Due to gauge invariance, only two phases are independent, therefore we set *ϕ*_M_ ≡ 0. Between lead L (R) and M, we assume a highly transmissive channel, fully described by two coupling parameters to the leads and hosting a spin-degenerate ABS, whose energy *E*_L(R)_ depends on *ϕ*_L(R)_ − *ϕ*_M_ ≡ *ϕ*_L(R)_. The choice of the coupling parameters led to transmissions *T*_1_ ≈ 0.998 and *T*_2_ ≈ 0.992 for the left and right ABS, respectively. Coupling between the ABSs is described by the parameter *t*, resulting in a minimal set of five numerical parameters, plus a broadening parameter (more details of the model are explained in Supplementary Note 1). When the two ABSs are isolated (*t* = 0), their energy dispersions solely depend on the transmission of the respective channels^2^. However, for finite coupling *t* ≠ 0, hybridisation between the states is enabled in the regions of (*ϕ*_L_, *ϕ*_R_) where the isolated energies *E*_L_ and *E*_R_ are comparable, resulting in an Andreev molecule^32,33^. To study the experimentally relevant case, we assume *t* = 1.1Δ, indicating ABSs strongly coupled to each other. In Fig. 3a, we display a numerical simulation of the Andreev energy bands as a function of *ϕ*_L_ and *ϕ*_R_ for negative energies (i.e., below the Fermi level *E*_F_ ≡ 0), noting that specular bands exist at positive energies owing to electron–hole symmetry. The finite coupling between the ABSs results in hybridised bands with a pronounced splitting. In the region with both phases tuned near to *π*, the band closer to zero energy shows a striking anisotropy in the phase space and the dispersion is strongly modified compared to that of high-transmission ABSs in a ballistic two-terminal JJ. To better compare experimental and numerical results, we calculate the DOS at fixed energy *E* = − 0.09Δ as a function of the cross-coupled phases ${\phi }_{{{{{{{{\rm{L}}}}}}}}}^{*}$ and ${\phi }_{{{{{{{{\rm{R}}}}}}}}}^{*}$ (Fig. 3b), and as a function of energy along the three phase space linecuts *γ*_1−3_ (Fig. 3c–e). All simulations qualitatively reproduce the key features observed in the measurements of Figs. 1d and 2, and lead to the same order of magnitude for the avoided crossings. In the constant-energy simulation (Fig. 3b), we confirm the presence of a periodic pattern characterised by avoided crossings and phase shifts of the ABS resonances near the intersection points, where individual ABSs are connected to each other forming closed loops. Further, the spectra presented in Fig. 3c–e resemble the results shown in Fig. 2d–f: the ABS dispersion approaching zero energy has a sharp peak along *γ*_1_ and is broader along *γ*_2_. The individual ABSs reappear with a significant separation when probed along *γ*_3_, while maintaining a sizeable avoided crossing. The experimentally measured phase shifts are larger than those calculated theoretically. The enhanced shift is likely produced by the finite inductive coupling between the loops, which is not included in the numerical model and implies a coupling between the fluxes Φ_L_ and Φ_R_. The change of slope of the ABS resonances between Fig. 1d, e is consistent with the phase shift and is related to the same coupling mechanism^50,51^. These effects are discussed in more detail in Supplementary Note 6.

### Tomography of the Andreev band structure

**Fig. 4:**
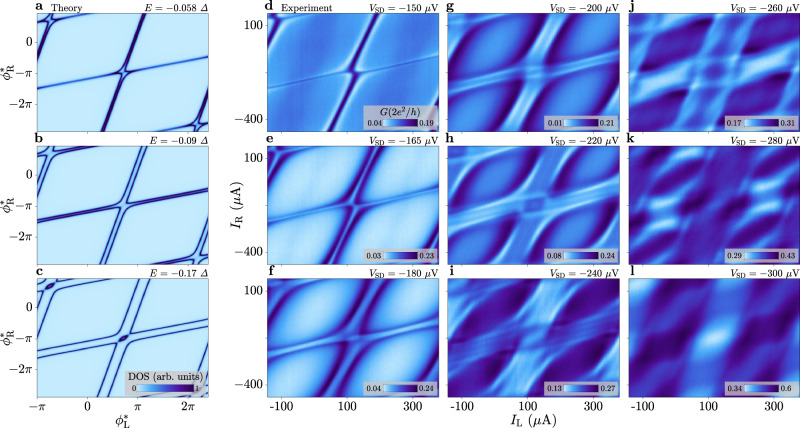
Constant-energy planes as a function of the two phases for different energies. **a**–**c** Simulated density of states as a function of the cross-coupled superconducting phase differences ${\phi }_{{{{{{{{\rm{L}}}}}}}}}^{*}$ and ${\phi }_{{{{{{{{\rm{R}}}}}}}}}^{*}$ at fixed values of energy *E*. **d**–**l** Differential tunnelling conductance *G* measured as a function of the currents *I*_L_ and *I*_R_ injected into the flux-bias lines at fixed values of voltage bias *V*_SD_, for *V*_Switch_ = 0.

Another visualisation of the ABS energy bands is provided by combining multiple constant-energy cut planes—each showing the dependence on both superconducting phase differences—to achieve a tomographic representation of the band structure. For this purpose, we measured the tunnelling conductance as a function of *I*_L_ and *I*_R_ for several values of *V*_SD_. The outcome is summarised in Fig. 4, where panels a–c display three theoretically calculated planes in the low-energy spectrum and d–l report experiments for nine values of *V*_SD_. At *V*_SD_ ≈ − Δ/*e* (i.e., near zero energy), where the ABSs are probed in proximity of their maximum, we observe single resonances (Fig. 4a, d), which split into pairs at more negative bias (Fig. 4b, e). We confirm the presence of the features described for Fig. 1d and Fig. 3b. Notably, in Fig. 4c, f, resonances related to different states are connected to each other by arcs enclosing an oval region. At even more negative bias, additional resonances arise, compatible with low-transmission ABSs appearing in the spectrum from *V*_SD_ ≈ −200 μV (see Fig. 2). Similar to the high-transmission ABSs discussed previously, low-transmission states first occur as single lines (Fig. 4g) and then split into pairs (Fig. 4h, i). Several additional modes emerge at larger ∣*V*_SD_∣, making it difficult to resolve individual states while approaching the continuum (Fig. 4j–l). We remark that the theoretical model assumes only two high-transmission modes and does not include any additional states with lower transmission, unlike the experimentally measured spectrum. Therefore, a direct comparison of the spectrum at high ∣*E*∣ is beyond the scope of the model.

Our main experimental findings, including avoided crossings and phase shifts in the constant-bias planes of Figs. 1d and 4, as well as the anisotropy highlighted in Figs. 2d, e and 3c, d, were qualitatively reproduced on a second device (see Supplementary Note 3). These measurements suggest the generality of the observed phenomena.

## Discussion

Supported by our theoretical model of coupled ABSs, we interpret the experimental results summarised in Figs. 1, 2 and 4 as evidence of coupling and hybridisation between two highly transmissive ABSs in the normal region of the 3TJJ. Thus, our devices constitute an implementation of an Andreev molecule, comparable to refs. ^32,33^. Avoided crossings in both the phase space and the energy spectrum, together with the anisotropic ABS dispersion motivate our interpretation. Unlike previous experimental studies of Andreev molecules in two-terminal geometries^52,53^, this realisation is in an open system and is a direct manifestation of the phase-controlled, multidimensional Andreev band structure.

Our system can be considered the magnetic dual of a double quantum dot^54^, where electric fields controlled by gate voltages, capacitance and charge on the dots (quantised in units of *e*) are substituted by magnetic fields controlled by currents in flux-bias lines, inductance and magnetic flux threading superconducting loops (quantised in units of the superconducting flux quantum Φ_0_ = *h*/2*e*, with *h* the Planck constant). Both in a double quantum dot and in the present Andreev molecule, overlapping wave functions of two discrete and localised states, coupling in a middle region, result in avoided crossings between their otherwise degenerate energy levels. In our device structure, two discrete levels, namely high-transmission ABSs, form in the short L–M and R–M junctions (whose minimum length is lithographically 30 nm) and are coupled to each other by their close proximity.

Spin-resolved ABSs are not observed in the experiments, well described by a spin degenerate model, despite the presence of spin–orbit coupling in our system. We note that here the spin–orbit length, *l*_SO_≈  150 nm for InAs^55^, is larger than the separation between pairs of terminals of the 3TJJ, resulting in a relatively weak strength of spin–orbit coupling. Enlarging the size of the 3TJJ would thus be required to resolve spin–orbit splitting of ABSs.

In conclusion, ABSs in hybrid 3TJJs were investigated with tunnelling spectroscopy measurements. Owing to the individual control over two superconducting phase differences, we explored a synthetic Andreev band structure and found signatures of coupling and hybridisation between two highly transmissive ABSs, consistent with their overlap in the 3TJJ region and the formation of an Andreev molecule. In the 2D phase space probed at constant voltage bias, we observed periodic patterns with avoided crossings and phase shifts near the intersections between ABS resonances. We measured the spectrum along selected linecuts of the phase space, finding a strong anisotropy of the ABS band structure and avoided crossings between the states. The experiments are well described by a theoretical model of two coupled ABSs. Our results provide new insights into the physics of multiterminal devices, establish phase control over the ABS band structure and demonstrate the feasibility of realising exotic Andreev matter. Future studies of multidimensional band structures could focus on phase-engineering spin-resolved Andreev levels^20^, ground state fermion parity transitions^20,56,57^ and topological bands, including Weyl singularities^21–25^.

*Note*. We recently became aware of the unpublished data of refs. ^58^ and ^59^, where three-terminal devices were investigated.

## Methods

### Materials and fabrication

Devices were fabricated in a III–V heterostructure grown with molecular beam epitaxy techniques on an InP (001) substrate. The stack consisted of a step-graded InAlAs buffer layer covered by an In_0.75_Ga_0.25_As/InAs/In_0.75_Ga_0.25_As quantum well and two monolayers of GaAs. The InAs layer, hosting a two-dimensional electron gas (2DEG), was 8 nm thick and buried 13 nm below the surface. On top of the III–V stack, a 15 nm thick Al layer was deposited in situ without breaking vacuum. Characterisation of the 2DEG in a gated Hall bar revealed a peak mobility of 18,000 cm^2^V^−1^s^−1^ at an electron sheet density of 8 × 10^11^cm^−2^. This resulted in an electron mean free path *l*_*e*_ ≳ 260 nm, indicating that both the three-terminal Josephson junction and the two-terminal switch junction were in the ballistic regime.

First, large mesa structures were isolated, suppressing parallel conduction between devices and across the middle regions of the superconducting loops. This was done by selectively etching the Al layer with Transene type D, followed by a second chemical etch to a depth of ≈ 380 nm into the III–V material stack, using a 220:55:3:3 solution of H_2_O:C_6_H_8_O_7_:H_3_PO_4_:H_2_O_2_. Next, Al was defined by wet etching with Transene type D at 50 °C for 4 s. The dielectric, deposited on the entire chip by atomic layer deposition, consisted of a 3-nm thick layer of ${{{{{{{{{\rm{Al}}}}}}}}}_{{{{{{{{\rm{2}}}}}}}}}}{{\rm{O}}}_{{{{{{{{\rm{3}}}}}}}}}$ and a 15-nm thick layer of HfO_2_. Gate electrodes and flux-bias lines were defined by evaporation and lift-off. In a first step, 5 nm of Ti and 20 nm of Au were deposited to realise the fine features of the gates; in a second step, a stack of Ti/Al/Ti/Au with thicknesses 5 nm, 340 nm, 5 nm and 100 nm was deposited to connect the mesa structure to the bonding pads and to define the flux-bias lines.

### Measurement techniques

Experiments were performed in a dilution refrigerator with base temperature at the mixing chamber below 10 mK. The sample was mounted on a QDevil QBoard sample holder system, without employing any light-tight enclosure. Electrical contacts to the devices, excepts for the flux-bias lines, were provided via a resistive loom with QDevil RF and RC low-pass filters at the mixing chamber stage, and RC low-pass filters integrated on the QBoard sample holder. Currents in the flux-bias lines were injected via a superconducting loom with only QDevil RF filters at the mixing chamber stage. Signals were applied to all gates and flux-bias lines via home-made RC filters at room temperature. Electrical transport measurements were performed with low-frequency AC lock-in techniques. A fixed AC voltage *δV*_SD_ = 5 μV at frequency 211 Hz and a variable DC voltage *V*_SD_ were applied to a contact at the superconducting probe (labelled S in Fig. 1a). The AC current *δI* and the DC current *I*_SD_ flowing in the grounded terminal D were measured via a current-to-voltage (I–V) converter. By measuring the AC voltage *δV* between terminals S and D in a four-terminal configuration, the differential conductance *G* ≡ *δI*/*δV* was determined. The refrigerator was equipped with a vector magnet which, despite not being utilised for the experiments, produced a small magnetic field offset. Hence, arbitrary offsets in the flux-bias line currents *I*_L_ and *I*_R_ of − 18 μA and 74 μA were considered in datasets, in such a manner that the point where *I*_L_ = *I*_R_ = 0 was at the centre of a diamond-like region in the constant-bias maps.

### Supplementary information

Supplementary Information

Peer Review

## Data Availability

The data presented in this study have been deposited in Zenodo [https://zenodo.org/record/8360770]. Further data that support the findings of this study are available upon request from the corresponding author.

## Code availability

Computer code used to perform the numerical simulations presented in this work has been deposited in Zenodo [https://zenodo.org/record/8360770].
