# Supplementary material for: Phase-engineering the Andreev band structure of a three-terminal Josephson junction
Source: Nat Commun. 2023 Oct 25;14:6784. doi: 10.1038/s41467-023-42356-6 (PMC10600130; doi:10.1038/s41467-023-42356-6)
Supplement: Supplementary file 1 — Supplementary Information [file 41467_2023_42356_MOESM1_ESM.pdf]

Supplementary Information

Phase-engineering the Andreev band structure of a three-terminal Josephson junction

Marco Coraiola,[1] Daniel Z. Haxell,[1] Deividas Sabonis,[1] Hannes Weisbrich,[2] Aleksandr E. Svetogorov,[2] Manuel Hinderling,[1] Sofieke C. ten Kate,[1] Erik Cheah,[3] Filip Krizek,[1,3,*] Rüdiger Schott,[3] Werner Wegscheider,[3] Juan Carlos Cuevas,[4] Wolfgang Belzig,[2] and Fabrizio Nichele[1,†]

[1] *IBM Research Europe—Zurich, 8803 Rüschlikon, Switzerland*

[2] *Fachbereich Physik, Universität Konstanz, D-78457 Konstanz, Germany*

[3] *Laboratory for Solid State Physics, ETH Zürich, 8093 Zürich, Switzerland*

[4] *Departamento de Física Teórica de la Materia Condensada and Condensed Matter Physics Center (IFIMAC), Universidad Autónoma de Madrid, E-28049 Madrid, Spain*

CONTENTS

Supplementary Note 1: Theory 2

Supplementary Note 2: Gate dependence of differential conductance 4

Supplementary Note 3: Results for Device 2 4

Supplementary Note 4: Results for different V_{G} 5

Supplementary Note 5: Mutual inductance matrix 5

Supplementary Note 6: Phase shifts and inductances in the system 6

Supplementary Figures 8

Supplementary References 15

* Present address: Institute of Physics, Czech Academy of Sciences, 162 00 Prague, Czech Republic

† fni@zurich.ibm.com

SUPPLEMENTARY NOTE 1: THEORY

In the following, we discuss hybridisation of two Andreev bound states (ABSs) between three superconducting leads. The problem has been investigated extensively in the context of the Andreev molecule [1–4]. Here, we treat hybridisation empirically with a coupling parameter γ introducing an avoided crossing in the spectrum. Assuming relatively weak coupling between the two ABSs, the spectrum resembles the energy levels of the independent states except for the points where they would cross, where hybridisation leads to the anticrossing. A simple perturbation theory can be applied if the energies $E_{\mathrm{L,R}}$ are sufficiently larger than γ:

$$E_{\pm} = \frac{E_{\mathrm{L}}(\phi_{\mathrm{L}}) + E_{\mathrm{R}}(\phi_{\mathrm{R}})}{2} \pm \sqrt{\left(\frac{\gamma}{2}\right)^2 + \left[\frac{E_{\mathrm{L}}(\phi_{\mathrm{L}}) - E_{\mathrm{R}}(\phi_{\mathrm{R}})}{2}\right]^2}, \tag{1}$$

where

$$E_\alpha = -\mathit{\Delta}\sqrt{1-\tau_\alpha^2 \sin^2\frac{\phi_\alpha}{2}}, \qquad \alpha \in \{\mathrm{L},\mathrm{R}\} \tag{2}$$

is the energy of an independent ABS between two superconducting leads with phase difference ϕ_α and energy gap $\mathit{\Delta}$ in a channel with transmission τ_α^2. We recall that the perturbation theory is applicable for $|E_\alpha(\phi_\alpha)| \gg |\gamma|$: this condition does not hold if the transmissions of the junctions are large ($1-\tau_\alpha \ll 1$) and the phase differences are close to $(2n+1)\pi$, which is the regime studied in the experiment. Nevertheless, in such experimentally relevant limit we follow the approach of Refs. [2, 3] and expand the independent ABS energies around $\phi_\alpha = \pi$:

$$E_\alpha \approx -\mathit{\Delta}\sqrt{1-\tau_\alpha^2\left[1-\frac{(\phi_\alpha-\pi)^2}{4}\right]} \approx -\frac{\mathit{\Delta}}{2}\sqrt{(\phi_\alpha-\pi)^2+(2r_\alpha)^2}, \tag{3}$$

with $r_\alpha^2 = 1-\tau_\alpha^2 \ll 1$ the reflection amplitude squared. In the limit of perfect transmission $r_{\mathrm{L}} = r_{\mathrm{R}} = 0$, the equations derived in Refs. [2, 3] for the ballistic regime can be employed:

$$E_{\pm} \approx -\frac{\mathit{\Delta}}{2}\sqrt{\delta^2 + \frac{(\varphi_{\mathrm{L}}+\varphi_{\mathrm{R}})^2}{4} + \frac{(\varphi_{\mathrm{L}}-\varphi_{\mathrm{R}})^2}{4} \pm (\varphi_{\mathrm{L}}-\varphi_{\mathrm{R}})\sqrt{\delta^2+\frac{(\varphi_{\mathrm{L}}+\varphi_{\mathrm{R}})^2}{4}}}, \tag{4}$$

where $\varphi_\alpha = \phi_\alpha - \pi$ and $\delta = \gamma/\mathit{\Delta}$ is the dimensionless coupling parameter. The formula is applicable for any points close to $(\phi_{\mathrm{L}}, \phi_{\mathrm{R}}) = [\pi(2n+1), \pi(2m+1)]$, with n and m integers. The corresponding constant-energy cut plane of the spectrum at $E = -0.01\mathit{\Delta}$ is shown in Supplementary Fig. 1a as a function of the phase differences. Here, hybridisation of purely ballistic channels introduces avoided crossings along the anti-diagonal direction $\phi_{\mathrm{L}} = 2\pi - \phi_{\mathrm{R}}$.

Moreover, we consider small but finite reflection amplitudes $r_\alpha \ll 1$ for each channel and use the effective Hamiltonian introduced in [2] (by projecting on the low-energy states) to derive the spectrum:

$$E_{\pm} \approx -\frac{\mathit{\Delta}}{2\sqrt{2}} \times$$
$$\times \sqrt{2\delta^2 + \varphi_{\mathrm{L}}^2 + (2r_{\mathrm{L}})^2 + \varphi_{\mathrm{R}}^2 + (2r_{\mathrm{R}})^2 \pm \sqrt{\left[\varphi_{\mathrm{L}}^2+(2r_{\mathrm{L}})^2-\varphi_{\mathrm{R}}^2-(2r_{\mathrm{R}})^2\right]^2 + 4\delta^2\left[(\varphi_{\mathrm{L}}-\varphi_{\mathrm{R}})^2+(2r_{\mathrm{L}})^2+(2r_{\mathrm{R}})^2\right]}}. \tag{5}$$

The constant-energy plane at $E = -0.01\mathit{\Delta}$ is displayed in Supplementary Fig. 1b, where we observe that non-zero reflection amplitudes introduce additional avoided crossings along the diagonal direction $\phi_{\mathrm{L}} = \phi_{\mathrm{R}}$.

In order to go beyond the limiting case accessible analytically, valid for weak coupling between the ABSs, we perform numerical simulations of a comparable system. Andreev states are modelled as two single-level quantum dots (QDs) coupled to three superconducting terminals. A coupling between the QDs accounts for hybridisation of the ABSs in the three-terminal junction. The model is schematically shown in Supplementary Fig. 2. For each ABS, the only relevant parameter is the transmission of the corresponding junction [5], hence we can arbitrarily opt for any microscopic model. The QD model offers a flexible tool to describe a scattering region and is well suited for numerical analyses of the system, even for strong coupling between the ABSs, where the analytical approach cannot be used. The coupling between each QD and the superconducting terminals can be arbitrarily strong, hence this model is also suited to describe ABSs in the open regime, rather than isolated dots weakly coupled to the leads [6].

The total Hamiltonian of the system is expressed as:

$$H = H_{\mathrm{DD}} + \sum_{\alpha=\mathrm{M,L,R}} H_{\mathrm{S},\alpha} + H_{\mathrm{DS}}. \tag{6}$$

Here, H_{DD} is the Hamiltonian of the double QD:

$$H_{\mathrm{DD}} = \epsilon_1 \sum_{\sigma} d^{\dagger}_{1\sigma} d_{1\sigma} + \epsilon_2 \sum_{\sigma} d^{\dagger}_{2\sigma} d_{2\sigma} + t \sum_{\sigma} d^{\dagger}_{1\sigma} d_{2\sigma} + \mathrm{h.c.}, \tag{7}$$

where the creation operator $d^{\dagger}_{j\sigma}$ of the j-th QD, its energy ϵ_j and the coupling parameter t are defined. The superconducting terminals are described by Bardeen–Cooper–Schriffer Hamiltonians [7]:

$$H_{\mathrm{S},\alpha} = \sum_{\boldsymbol{k}\sigma} \xi_{\boldsymbol{k}} c^{\dagger}_{\boldsymbol{k}\sigma,\alpha} c_{\boldsymbol{k}\sigma,\alpha} + \sum_{\boldsymbol{k}} \left(\mathit{\Delta} \mathrm{e}^{i\phi_\alpha} c^{\dagger}_{\boldsymbol{k}\uparrow,\alpha} c^{\dagger}_{-\boldsymbol{k}\downarrow,\alpha} + \mathit{\Delta} \mathrm{e}^{-i\phi_\alpha} c_{-\boldsymbol{k}\downarrow,\alpha} c_{\boldsymbol{k}\uparrow,\alpha} \right), \tag{8}$$

where $c^{\dagger}_{\boldsymbol{k}\sigma,\alpha}$ is the creation operator of an electron with momentum $\boldsymbol{k}$ and spin σ in lead $\alpha \in \{\mathrm{M, L, R}\}$, $\mathit{\Delta}$ is the superconducting gap (assumed to be the same for all leads), ϕ_α the superconducting phase of lead α and $\xi_{\boldsymbol{k}}$ the normal state dispersion in the leads. The tunnel coupling between each QD and the superconductors is expressed by the term H_{DS}, having the form:

$$H_{\mathrm{DS}} = v_{\mathrm{M},1} \sum_{\boldsymbol{k},\sigma} d^{\dagger}_{1\sigma} c_{\boldsymbol{k}\sigma,\mathrm{M}} + v_{\mathrm{M},2} \sum_{\boldsymbol{k},\sigma} d^{\dagger}_{2\sigma} c_{\boldsymbol{k}\sigma,\mathrm{M}} + v_{\mathrm{L},1} \sum_{\boldsymbol{k},\sigma} d^{\dagger}_{1\sigma} c_{\boldsymbol{k}\sigma,\mathrm{L}} + v_{\mathrm{R},2} \sum_{\boldsymbol{k},\sigma} d^{\dagger}_{2\sigma} c_{\boldsymbol{k}\sigma,\mathrm{R}} + \mathrm{h.c.}, \tag{9}$$

where $v_{\alpha,j}$ denotes the coupling of the j-th QD to lead α.

Two superconductor–QD–superconductor junctions are identified: between leads L and M through QD_1 and between leads R and M through QD_2. In the absence of coupling between the QDs ($t = 0$), the ABS energy of $\mathrm{QD}_{1(2)}$ depends only on $\phi_{\mathrm{L(R)}} - \phi_{\mathrm{M}}$. For finite coupling ($t \neq 0$) the ABSs hybridise, which is the case presented in the Main Text. We do not consider a direct ballistic channel between terminals L and R due to their larger separation in the device compared to the L–M and R–M junctions. Nevertheless, the energy of a hypothetical ABS in such channel would depend on $\phi_{\mathrm{L}} - \phi_{\mathrm{R}}$, hence it would be close to the superconducting gap in proximity of the points where $\phi_{\mathrm{L}} = \phi_{\mathrm{R}} = \pi$. As a consequence, any hybridisation of the additional state with the two included in the model would be suppressed near those regions of phase space. To evaluate this model in the limit of strong coupling between superconducting leads and QDs, i.e., $\mathit{\Gamma}_{\alpha,j} \equiv \pi N_0 v^2_{\alpha,j} > \mathit{\Delta}$ (where N_0 is the normal density of states in the leads), we calculate the ABS density of states by determining the Green's function of the coupled system with the Dyson equation:

$$\boldsymbol{G}_{\mathrm{DD}} = \boldsymbol{g}_{\mathrm{DD}} + \boldsymbol{g}_{\mathrm{DD}} \boldsymbol{V}_{\mathrm{DS}} \boldsymbol{g}_{\mathrm{SS}} \boldsymbol{V}^{\dagger}_{\mathrm{DS}} \boldsymbol{G}_{\mathrm{DD}}, \tag{10}$$

where $\boldsymbol{G}_{\mathrm{DD}}$ is the dressed Green's function of the double QD, $\boldsymbol{g}_{\mathrm{DD}}$ the unperturbed Green's function of the two QDs, $\boldsymbol{V}_{\mathrm{DS}}$ the coupling between the QDs and the superconducting leads and $\boldsymbol{g}_{\mathrm{SS}} = \mathrm{diag}\left(\boldsymbol{g}_{\mathrm{SS,M}}, \boldsymbol{g}_{\mathrm{SS,L}}, \boldsymbol{g}_{\mathrm{SS,R}}\right)$ the unperturbed Green's function of the three leads. From this expression, the ABS density of states is calculated as $\rho = -\frac{1}{\pi}\mathrm{Im}\left\{\mathrm{tr}\left(\boldsymbol{G}_{\mathrm{DD}}\right)\right\}$ and the eigenenergies are obtained from the poles of $\boldsymbol{G}_{\mathrm{DD}}$, yielding the results shown in Figs. 3 and 4 of the Main Text. As previously discussed, the only relevant parameters for the hybridised ABSs are the inter-QD coupling t and the transmission amplitudes, which are related to the QD–lead couplings $\mathit{\Gamma}_{\alpha,j}$ via the expressions:

$$T_1 = \frac{4\,\mathit{\Gamma}_{\mathrm{M},1}\,\mathit{\Gamma}_{\mathrm{L},1}}{\left(\mathit{\Gamma}_{\mathrm{M},1} + \mathit{\Gamma}_{\mathrm{L},1}\right)^2}, \qquad T_2 = \frac{4\,\mathit{\Gamma}_{\mathrm{M},2}\,\mathit{\Gamma}_{\mathrm{R},2}}{\left(\mathit{\Gamma}_{\mathrm{M},2} + \mathit{\Gamma}_{\mathrm{R},2}\right)^2}, \tag{11}$$

valid in the limit of strong lead–QD coupling. These parameters influence the region of the avoided crossing between the two ABS, as illustrated in Supplementary Fig. 3. In all simulations, a broadening parameter η was assumed in the Green's functions. We observe qualitative agreement between the numerical simulations and the analytical study previously discussed (see Supplementary Fig. 1).

The parameters used for the simulations in the Main Text were: $t = 1.1\mathit{\Delta}$, $\mathit{\Gamma}_{\mathrm{L},1} = 5.5\mathit{\Delta}$, $\mathit{\Gamma}_{\mathrm{M},1} = \mathit{\Gamma}_{\mathrm{R},2} = 6\mathit{\Delta}$, $\mathit{\Gamma}_{\mathrm{M},2} = 5\mathit{\Delta}$, corresponding to transmissions $T_1 \approx 0.998$ and $T_2 \approx 0.992$, and broadening $\eta = 0.02\mathit{\Delta}$. Moreover, we recall that the constant-energy planes of Figs. 3b and 4a–c were plotted by introducing a cross-dependence between the phase differences $\phi_{\mathrm{L}} - \phi_{\mathrm{M}} \equiv \phi_{\mathrm{L}}$ and $\phi_{\mathrm{R}} - \phi_{\mathrm{M}} \equiv \phi_{\mathrm{R}}$ to better represent the experimental data, where a cross-dependence between the two flux-bias lines was present (see also discussion in Section). In particular, the cross-coupled phases ϕ^*_{L} and ϕ^*_{R} were defined as linear combinations of ϕ_{L} and ϕ_{R} with the transformation:

$$\begin{pmatrix} \phi^*_{\mathrm{L}} \\ \phi^*_{\mathrm{R}} \end{pmatrix} = \begin{pmatrix} a & b \\ c & d \end{pmatrix} \cdot \begin{pmatrix} \phi_{\mathrm{L}} \\ \phi_{\mathrm{R}} \end{pmatrix}, \tag{12}$$

where the coefficients $a = 0.9708$, $b = 0.2400$, $c = 0.2832$ and $d = 1.122$ were used.

SUPPLEMENTARY NOTE 2: GATE DEPENDENCE OF DIFFERENTIAL CONDUCTANCE

The transmission between the superconducting probe and the three-terminal Josephson junction (3TJJ) was controlled with two gate electrodes, denoted tunnel gates and set to the same voltage $V_\mathrm{T} \equiv V_\mathrm{TL} = V_\mathrm{TR}$, and had a finite dependence on the gates energised by the voltages V_Probe and V_G, denoted probe gate and global gate respectively (see Fig. 1a,c). We always kept $V_\mathrm{Probe} = 150$ mV. The effect of the gate at voltage V_Switch (switch gate) on the probe transmission was negligible. The differential conductance G measured as a function of V_T and V_SD, with V_SD the DC voltage bias applied to the probe, is shown in Supplementary Fig. 4a,b for $V_\mathrm{G} = 50$ mV and $V_\mathrm{G} = -150$ mV respectively. In both cases the conductance, hence the probe transmission, decreases with V_T, transitioning from open to tunnelling regime. In the former, we observe a zero-bias conductance peak, corresponding to a remnant of supercurrent between the probe and the superconducting leads, and several peaks at finite bias, related to multiple Andreev reflection (MAR) processes. In the tunnelling regime, where the conductance at large $|V_\mathrm{SD}|$ is substantially lower than the conductance quantum $G_0 = 2e^2/h$, a transport gap of ≈ 310 μV is present in the spectrum, consistent with a superconducting gap of Al $\varDelta \approx 155$ μeV. Pronounced features appearing at the edges of the gap correspond to Andreev bound states (ABSs). As shown in Supplementary Fig. 4b, by further lowering V_T the probe could be completely pinched off. We remark that tunnelling spectroscopy in our devices was performed in a superconductor–insulator–superconductor (SIS) configuration, hence the $G(V_\mathrm{SD})$ traces result from a convolution product between two relatively complex densities of states [8, 9] and do not provide a direct measurement of the gap hardness. Nevertheless, since $G \approx 0$ across a significant voltage range around $V_\mathrm{SD} = 0$ (for any flux-line currents I_L and I_R, as seen in Fig. 2 of the Main Text), we consider the gap hardness to be comparable to previous reports [10], where also a density-of-states broadening to what we noted in the Main Text was observed.

The dependence on the global gate is presented in Supplementary Fig. 4c for $V_\mathrm{T} = -1.07$ V. We find that its main effect on the spectrum is also to change the transmission of the probe, which is progressively reduced for decreasing V_G until the pinch off is reached. Notably, in both the V_T and the V_G dependence, no sharp conductance peaks relatable to resonant transport via spurious quantum dots are observed.

Tunnelling spectroscopy measurements were performed on a second device, fabricated on the same chip of the first and measured in the same cool down. Device 2 was lithographically similar to Device 1, except for the width of the superconducting probe and the shape of the tunnel and probe gates (see Supplementary Fig. 4d). The dependences on the tunnel gate voltage V_T and on the global gate voltage V_G are plotted in Supplementary Fig. 4e and f respectively, showing features qualitatively very similar to Device 1.

SUPPLEMENTARY NOTE 3: RESULTS FOR DEVICE 2

The main experimental results shown in the Main Text for Device 1 were qualitatively reproduced in Device 2, as illustrated in Supplementary Figs. 5 and 6. Here, the tunnel gates were set to $V_\mathrm{T} = -1.395$ V and $V_\mathrm{T} = -1.42$ V respectively, with the global gate voltage set to $V_\mathrm{G} = -150$ mV and the probe gate voltage to $V_\mathrm{Probe} = 100$ mV. In these configurations, the probe was in the tunnelling regime and its transmission was comparable to that of Device 1 for the measurements presented in the Main Text.

In Supplementary Fig. 5, we show the constant-bias planes (i.e., G as a function of I_L and I_R at fixed values of V_SD) corresponding to Figs. 1d,e, and 4d–l of the Main Text. When the switch junction is in the ON state (defined by $V_\mathrm{Switch} = 0$), we confirm the presence of avoided crossings between a $\varPhi_\mathrm{L}$-dependent ABS and a $\varPhi_\mathrm{R}$-dependent ABS, with the resonances associated to one state connecting to those of the other, and phase shifts occurring near the avoided crossings. The 2D pattern is strongly simplified when the switch is OFF ($V_\mathrm{Switch} = -1.5$ V, Supplementary Fig. 5b), as the $\varPhi_\mathrm{R}$-dependent ABS disappears and no sign of hybridization is observed. The band structure tomography is displayed in Supplementary Figs. 5c–k and is compatible with the results presented in the Main Text (see Fig. 4).

Further, we select linecuts of the phase space, indicated by the coloured arrows in Supplementary Figs. 5a,b, along which we perform bias-dependent spectroscopy (Supplementary Fig. 6, to be compared with Fig. 2). Again, we observe strong dispersion anisotropy when comparing linecut γ_1 to γ_2. Incidentally, while we still note a conductance peak at $V_\mathrm{SD} = \pm 155$ μV, whose position in bias does not vary appreciably with γ_i and which is attributed to MAR processes, this device does not reveal a second peak at larger $|V_\mathrm{SD}|$. This shows that the peak at $V_\mathrm{SD} = \pm 175$ μV in Device 1 is a device-specific feature. Since the ABS dispersion of Device 1 is qualitatively reproduced in Device 2, we corroborate that the peak at ± 175 μV in the former does not interact with the ABSs or affect their main properties.

SUPPLEMENTARY NOTE 4: RESULTS FOR DIFFERENT V_G

The measurements of Device 1 shown in the Main Text were obtained in a single gate configuration (except for the switch gate voltage, set to −1.5 V for Figs. 1e, 2i and to 0 V elsewhere). The main results were reproduced in different gate configurations, defined by setting a new value of V_{G} and adjusting V_{T} to maintain a comparable probe transmission, crucially remaining in the tunnelling regime. In Supplementary Figs. 7–12 we show the three cases $V_{\mathrm{G}} = -66$ mV, $V_{\mathrm{G}} = -150$ mV and $V_{\mathrm{G}} = -250$ mV, as described in the following. Remarkably, all the key features discussed in the Main Text were qualitatively reproduced in these configurations, highlighting the generality of our results and further supporting our conclusions. Incidentally, we did not observe a variation in the transmission of the highly transmissive ABSs as a function of the global gate voltage, namely they approached the edges of the transport gap at $|V_{\mathrm{SD}}| \approx 155\ \mu$V very closely for any V_{G}. This was verified in the range -300 mV $\leq V_{\mathrm{G}} \leq 150$ mV, noting that at $V_{\mathrm{G}} = -300$ mV the sub-gap states were at a limit of visibility but still preserved the same dispersion with phase.

- $V_{\mathrm{G}} = -66$ mV: Supplementary Figs. 7a,b, 7c–k and 8 correspond to Figs. 1d,e, 4 and 2a–h respectively. V_{T} is adjusted to −1.038 V. Interestingly, we observe that the two ABSs have very similar visibility in this regime (namely, the resonances associated with each state have similar conductance), in contrast to the regime shown in the Main Text ($V_{\mathrm{G}} = 50$ mV), where the ABS dispersing with Φ_{L} had higher conductance. As a possible explanation, we hypothesise that, when V_{G} is reduced, the electrostatic potential profile at the tunnelling barrier shifts slightly, in such a manner that its maximum displaces from left to right. At the optimised value $V_{\mathrm{G}} = -66$ mV, transport between the probe and either side of the three-terminal region is symmetric, resulting in similar conductance when tunnelling into either ABS. Furthermore, in the constant-bias planes of Supplementary Fig. 7, and particularly in panel b, we notice an additional set of resonances with the same dependence on I_{L} and I_{R} as the left ABS. This is attributed to a very small phase modulation of the MAR peak, i.e., the nearly flat line at $V_{\mathrm{SD}} = -155\ \mu$V that is visible in Supplementary Fig. 8. These peak, particularly prominent in the present regime, accounts for a conductance background in the constant-bias maps and its modulation translates into a phase-periodic background.
- $V_{\mathrm{G}} = -150$ mV: Supplementary Figs. 9a,b, 9c–k and 10 correspond to Figs. 1d,e, 4 and 2 respectively. V_{T} is adjusted to −1.02 V, where the probe transmission is smaller than in the previous regimes: the differential conductance at large V_{SD} (near the range limits) is reduced by approximately a factor 2. This results in a lower signal-to-noise ratio, due to which periodic noise features become visible in the constant-bias measurements (Supplementary Fig. 9), as long as G is relatively small. At the same time, the conductance resonances at $V_{\mathrm{SD}} = \pm 155\ \mu$V and $V_{\mathrm{SD}} = \pm 175\ \mu$V in the spectra of Supplementary Fig. 10 become substantially less prominent, and so is their modulation as a function of γ_i. Visibly, the ABS dispersion is not distorted when the states cross the horizontal peaks, remaining qualitatively very similar to that observed in Fig. 2. This provides further support to the absence of an interaction between the peaks at $V_{\mathrm{SD}} = \pm 155\ \mu\mathrm{V}, \pm 175\ \mu$V and the ABSs. Finally, in this gate configuration, the ABS depending on Φ_{R} shows the highest conductance, consistent with the argument presented for the case $V_{\mathrm{G}} = -66$ mV.
- $V_{\mathrm{G}} = -250$ mV: Supplementary Figs. 11a,b, 11c–k and 12 correspond to Figs. 1d,e, 4 and 2a,b,d,e,i respectively. V_{T} is adjusted to −984 mV, where the probe transmission is comparable to the regime $V_{\mathrm{G}} = -150$ mV.

SUPPLEMENTARY NOTE 5: MUTUAL INDUCTANCE MATRIX

As described in the Main Text, our devices feature two flux bias lines where currents I_{L} and I_{R} are injected. Since I_{L} generates a magnetic field that is stronger over the left superconducting loop than over the right one, it controls mainly the external magnetic flux Φ_{L} threading the left loop, associated to the superconducting phase difference between the terminals L and M (see Fig. 1a). Similarly, I_{R} tunes mostly Φ_{R}, thus the phase difference between R and M, in such a manner that the combination of the two flux lines enables full phase control over a two-dimensional space. Nevertheless, each flux line has also a finite coupling to the opposite loop, thus Φ_{L} and Φ_{R} depend on both I_{L} and I_{R} according to the linear relation:

$$\begin{pmatrix} \Phi_{\mathrm{L}} \\ \Phi_{\mathrm{R}} \end{pmatrix} = \mathbf{M} \cdot \begin{pmatrix} I_{\mathrm{L}} \\ I_{\mathrm{R}} \end{pmatrix} = \begin{pmatrix} M_{\mathrm{LL}} & M_{\mathrm{LR}} \\ M_{\mathrm{RL}} & M_{\mathrm{RR}} \end{pmatrix} \cdot \begin{pmatrix} I_{\mathrm{L}} \\ I_{\mathrm{R}} \end{pmatrix}, \tag{13}$$

where $\mathbf{M}$ is the mutual inductance matrix. We calculate $\mathbf{M}$ from the constant-bias conductance measurement of Fig. 1d, plotted again in Supplementary Fig. 13a. First, we observe that the origin $(I_{\mathrm{L}}, I_{\mathrm{R}}) = (0, 0)$ corresponds to the origin of the flux space $(\Phi_{\mathrm{L}}, \Phi_{\mathrm{R}}) = (0, 0)$. We associate the centres of the adjacent diamond-like regions to the

addition or subtraction of one superconducting flux quantum $\Phi_0 = h/(2e)$ to either Φ_L or Φ_R. By evaluating Eq. 13 in two centre points, for example those related to $(\Phi_0, 0)$ and $(0, \Phi_0)$, we write a 4×4 equation system and determine:

$$\mathbf{M} = \begin{pmatrix} 6.96 \text{ pH} & -1.43 \text{ pH} \\ -1.69 \text{ pH} & 5.79 \text{ pH} \end{pmatrix}. \tag{14}$$

The ratio $M_{RR}/M_{LL} \approx M_{LR}/M_{RL} \approx 0.8$ approximately corresponds to the ratio between the length of the vertical segments of the right and left flux line. Knowing $\mathbf{M}$, we can apply the linear transformation of Eq. 13 to convert the (I_L, I_R) axes to the (Φ_L, Φ_R) axes, as illustrated in Supplementary Fig. 13b for the dataset of panel a.

SUPPLEMENTARY NOTE 6: PHASE SHIFTS AND INDUCTANCES IN THE SYSTEM

In the constant-bias measurements showing the dependence on I_L and I_R with the switch junction ON, such as in Figs. 1d and 4d–i, we remarked the presence of phase shifts occurring when resonances associated to different states (i.e., the Φ_L- and the Φ_R-dispersing ABS) intersect each other. Concomitantly, we noted a slope variation of the Φ_L-dispersing ABS depending on the ON/OFF state of the switch junction (yellow dashed line in Fig. 1e). In this section, we quantify the shifts and relate them to the different inductive contributions in the device. We find that a Josephson-like mutual inductive coupling between the two loops accounts for the presence of both the shifts and the slope difference.

Since a shift is present for both ABSs, it corresponds to a vector $(\Delta\Phi_L,\ \Delta\Phi_R)$ in the space of the two external magnetic fluxes Φ_L and Φ_R. For the extraction of this vector, we consider again the dataset of Fig. 4e from the Main Text, shown also in Supplementary Fig. 13c and, upon applying the basis transformation described in Section , in Supplementary Fig. 13d as a function of Φ_L and Φ_R. As a guide for the eye, we overlay lines following the dips between pairs of ABS resonances. In panel c, we mark the (vector) shifts $\mathbf{\Delta I}^*_L$ and $\mathbf{\Delta I}^*_R$ along the periodicity directions, while in panel d the shifts $\Delta\Phi_L$ and $\Delta\Phi_R$ are by construction parallel to the axes. These quantities are linked by the following relations:

$$\begin{pmatrix} \Delta\Phi_L \\ 0 \end{pmatrix} = \mathbf{M} \cdot \mathbf{\Delta I}^*_L, \qquad \begin{pmatrix} 0 \\ \Delta\Phi_R \end{pmatrix} = \mathbf{M} \cdot \mathbf{\Delta I}^*_R. \tag{15}$$

From the data, we find $\Delta\Phi_L \approx \Delta\Phi_R \approx 0.092 \cdot \Phi_0 \equiv \Delta\Phi$, showing that the effect is symmetric for the two ABSs.

This phase shift is then expressed as $\Delta\Phi = M_{cpl} \cdot I_{circ}$, namely as the product between a mutual inductance, accounting for the coupling between two fluxes, and a circulating current in either loop. Neither M_{cpl} nor I_{circ} can be directly determined from the experimental data (only their product $\Delta\Phi$), therefore we estimate the inductive coupling term considering different origins. The geometric mutual inductance between the two loops of our devices is $M_{geom} \approx 7.3$ pH [11]. Since the loops share a strip of epitaxial Al, its inductance, dominated by the kinetic inductance L_k, has to be added to M_{geom} [12, 13]. The kinetic inductance of the strip is estimated as [14]:

$$L_k = \frac{l}{w}\frac{h}{2\pi^2}\frac{R_\square}{\Delta} \approx 44 \text{ pH}, \tag{16}$$

where $l = 25$ μm and $w = 1$ μm are the length and width of the strip, $R_\square \approx 1.5$ Ω the normal state resistivity of the heterostructure stack measured in a Hall bar geometry (where the Al film was not removed) and $\Delta \approx 180$ μeV the superconducting gap of Al. The geometric and kinetic contribution lead to a combined mutual inductance $M_{loops} \approx 51.3$ pH. If we assume that $M_{cpl} = M_{loops}$, we require a circulating current $I_{circ} \approx 3.7$ μA for the shift $\Delta\Phi = 0.092 \cdot \Phi_0$. However, this value is much larger than any reasonable estimate of the critical current between each pair of terminals, which constitutes an upper bound to the supercurrent circulating in the loops. As a consequence, M_{cpl} must be substantially larger than M_{loops} to limit I_{circ}.

The only contribution that we have not discussed so far is a Josephson-like coupling due to the 3TJJ. Based on its geometry, we expect our 3TJJ to have a critical current of the order of 100 nA and a Josephson inductance of a few nH, which would explain the phase shift to a good degree. We deduce that, in our devices, the phase shifts derive from a Josephson-like coupling term rather than the geometric and kinetic inductive couplings, in analogy with existing couplers between superconducting qubits containing Josephson elements [15, 16].

This mutual coupling between Φ_L and Φ_R is also responsible for the slope difference depending on the state of the switch junction (see Fig. 1d,e). In the OFF state, no circulating current can flow in the right loop, thus no mutual effect is present. In the (I_L, I_R) plane (Fig. 1e of the Main Text), the ABS resonance is parallel to the Φ_R axis (indicated in Fig. 1d). When the switch junction is ON, the slope of the ABSs deviates from the directions of Φ_L and Φ_R. This is clearly visible on the (Φ_L, Φ_R) axes (Supplementary Fig. 13b,d), as the resonances are not vertical

or horizontal. In fact, due to the mutual coupling, the variation of a flux (for example Φ_{L}) from 0 to $\Phi_0/2$ causes a gradual change in the other (Φ_{R}), up to a maximum shift. Upon crossing $\Phi_{\mathrm{L}} = \Phi_0/2$, the circulating current in the left loop reverses direction, consequently the induced current in the right loop and the Φ_{R}-shift also flip sign. Therefore, the finite ABS slopes on the (Φ_{L}, Φ_{R}) axes (corresponding to the slope difference depending on the state of the switch junction) and the phase shifts are consistent. Both result from the finite Josephson-like mutual inductive coupling between the two loops.

SUPPLEMENTARY FIGURES

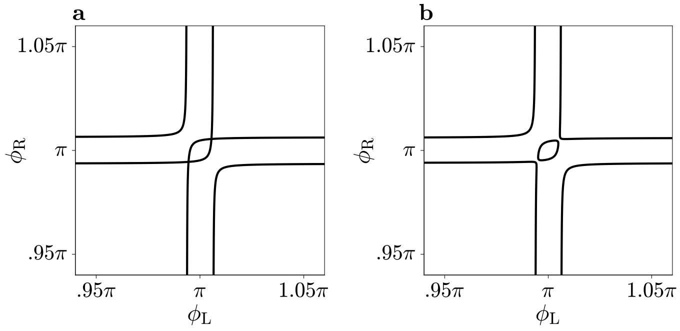

Supplementary Fig. 1. **Analytical calculations. a**,**b**, Constant-energy planes at $E = -0.01\Delta$ as a function of the superconducting phase differences ϕ_L and ϕ_R for coupling $\delta = 0.01$, in the fully ballistic regime $r_L = r_R = 0$ (**a**) and with finite normal reflection amplitudes $r_L = 0.003$, $r_R = 0.002$ (**b**).

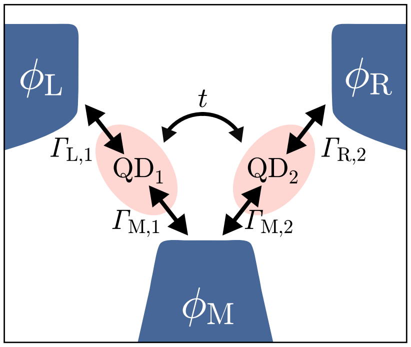

Supplementary Fig. 2. **Schematic of the numerical model.** Andreev bound states in the three-terminal Josephson junction are modelled as two single-level quantum dots (QDs) coupled to three superconducting leads with phases ϕ_L, ϕ_R and ϕ_M. $QD_{1(2)}$ is coupled to leads L (R) and M, as represented by the parameters $\Gamma_{L,1}$ and $\Gamma_{M,1}$ ($\Gamma_{R,2}$ and $\Gamma_{M,2}$) respectively. The parameter t accounts for the coupling between the QDs, enabling their hybridisation.

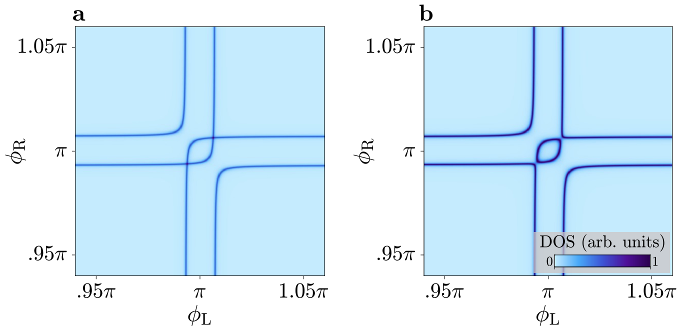

Supplementary Fig. 3. **Numerical simulations. a**,**b**, Density of states at fixed energy $E = -0.01\Delta$ as a function of the superconducting phase differences $\phi_L - \phi_M \equiv \phi_L$ and $\phi_R - \phi_M \equiv \phi_R$, for coupling energy $t = 0.05\Delta$ and broadening $\eta = 5{\cdot}10^{-4}\Delta$. In **a**, $\Gamma_{M,1} = \Gamma_{M,2} = \Gamma_{L,1} = \Gamma_{R,2} = 5\Delta$, namely perfect transmissions are assumed. In **b**, $\Gamma_{L,1} = 4.98\Delta$, $\Gamma_{M,1} = \Gamma_{R,2} = 5\Delta$ and $\Gamma_{M,2} = 4.97\Delta$, hence finite reflection amplitudes are considered. In both cases, the effective coupling $t/\Gamma \approx 0.01$ is similar to that introduced for the analytical case (see Fig. 1).

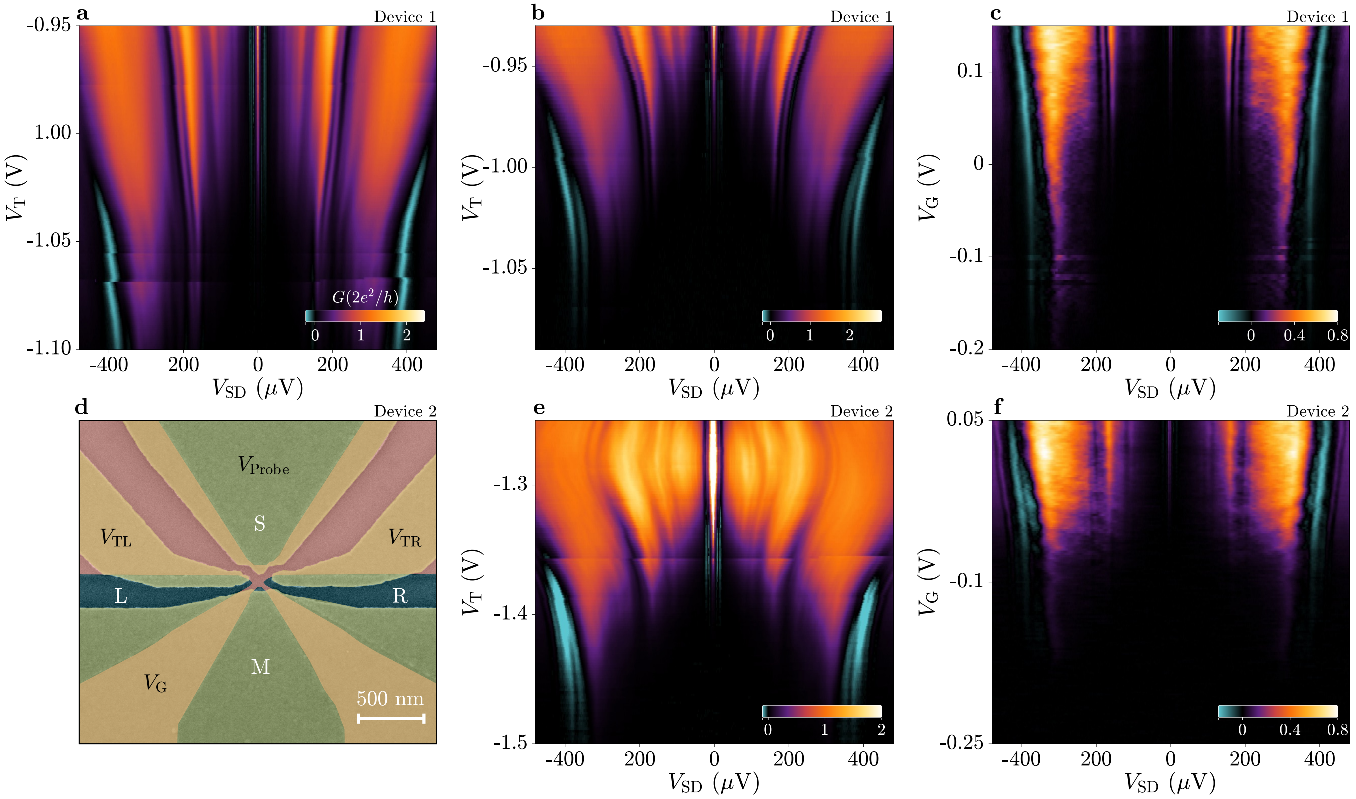

Supplementary Fig. 4. **Gate dependence of differential conductance.** **a**,**b**, Differential conductance G as a function of voltage bias V_{SD} and tunnelling gates voltage $V_T \equiv V_{TL} = V_{TR}$ in Device 1, for global gate voltage $V_G = 50$ mV in **a** and $V_G = -150$ mV in **b**. **c**, G as a function of V_{SD} and V_G in Device 1, for $V_T = -1.07$ V. **d**, False-coloured scanning electron micrograph of Device 2 in proximity of the three-terminal junction region (see Fig. 1a–c of the Main Text for the colour legend). **e**, G as a function of V_{SD} and V_T in Device 2, for $V_G = -150$ mV and $V_{Probe} = 100$ mV. **f**, G as a function of V_{SD} and V_G in Device 2, for $V_T = -1.5$ V and $V_{Probe} = 100$ mV.

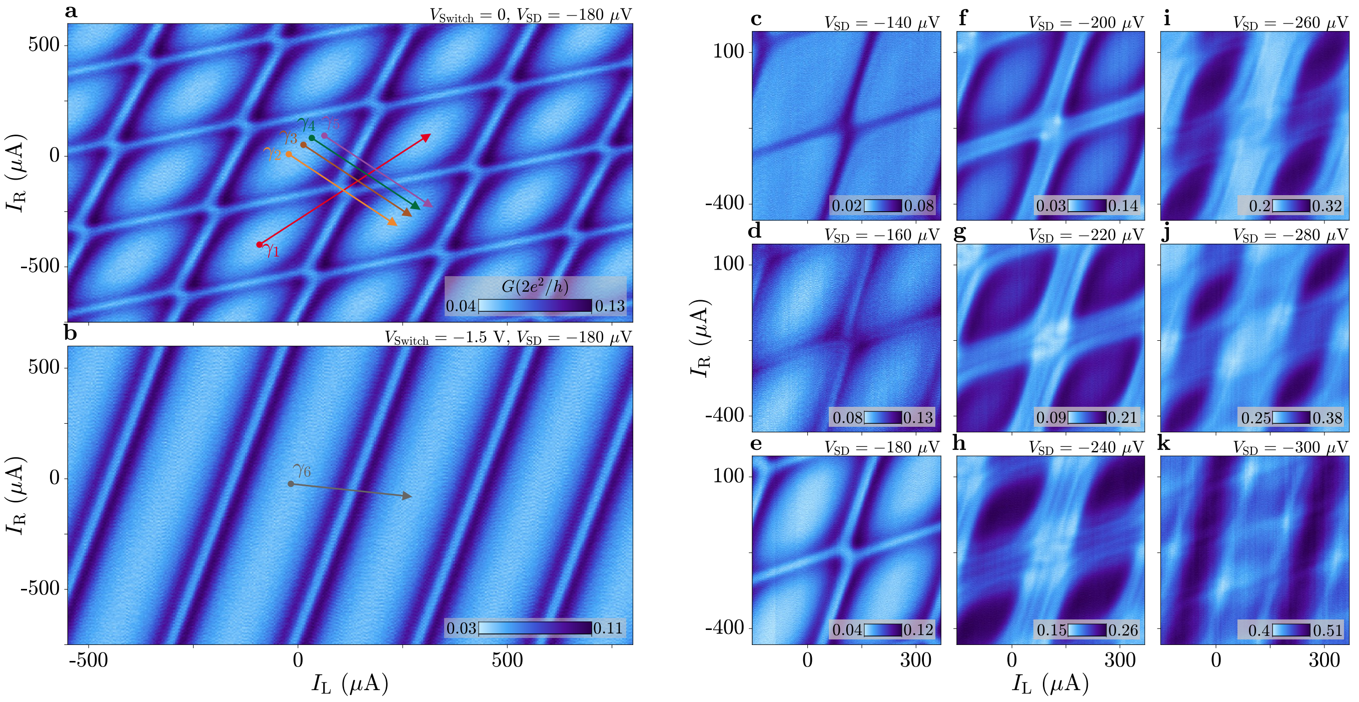

Supplementary Fig. 5. **Constant-bias planes as a function of the two phases in Device 2. a–k**, Differential conductance G as a function of the currents $I_{\rm L}$ and $I_{\rm R}$ injected into the flux-bias lines at fixed values of voltage bias $V_{\rm SD}$. **a**, $V_{\rm Switch}=0$, $V_{\rm SD}=-180\ \mu$V. **b**, $V_{\rm Switch}=-1.5$ V, $V_{\rm SD}=-180\ \mu$V. **c–k**, $V_{\rm Switch}=0$, $V_{\rm SD}$ varied between $-140\ \mu$V (**c**) and $-300\ \mu$V (**k**).

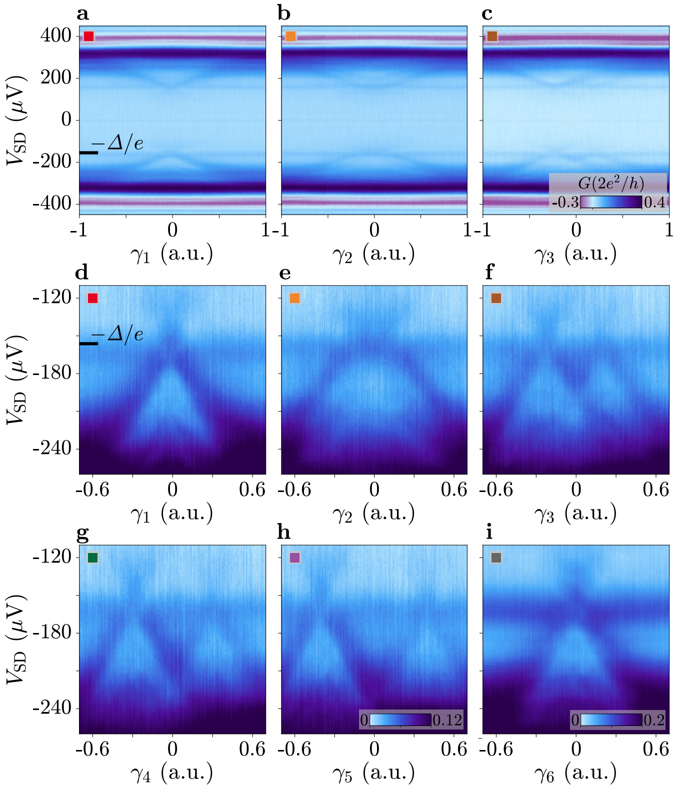

Supplementary Fig. 6. **Tunnelling conductance along phase space linecuts in Device 2. a–c**, Differential conductance G as a function of voltage bias $V_{\rm SD}$ along the linecuts γ_i (coloured arrows in Fig. 5a), for $V_{\rm Switch}=0$. **d–h**, As **a–c**, but plotted over restricted ranges of $V_{\rm SD}$ and γ_i. **i**, As **d–h**, but along linecut γ_6 (defined in Fig. 6), for $V_{\rm Switch}=-1.5$ V. The colourbar in **h** applies to **d–h**.

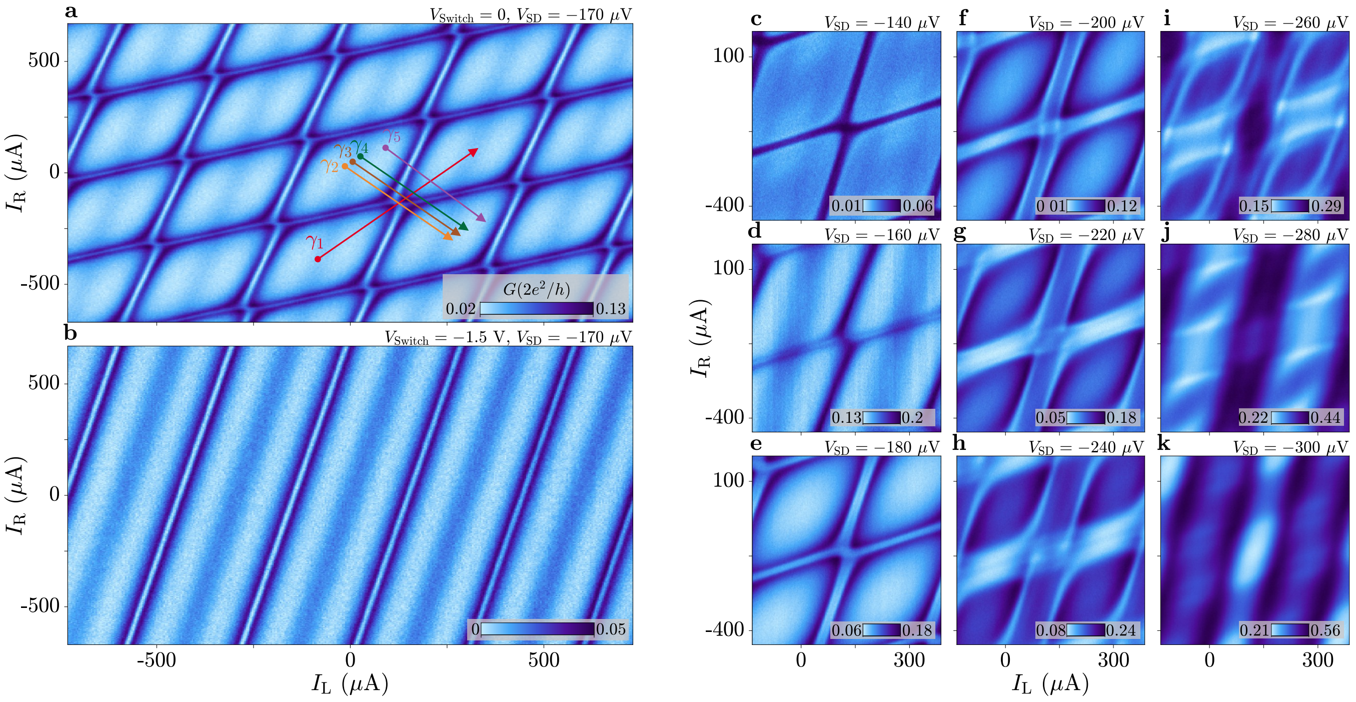

Supplementary Fig. 7. **Constant-bias planes as a function of the two phases at $V_{\rm G} = -66$ mV. a–k**, Differential conductance G as a function of the currents $I_{\rm L}$ and $I_{\rm R}$ injected into the flux-bias lines at fixed values of voltage bias $V_{\rm SD}$. **a**, $V_{\rm Switch} = 0$, $V_{\rm SD} = -170\ \mu$V. **b**, $V_{\rm Switch} = -1.5$ V, $V_{\rm SD} = -170\ \mu$V. **c–k**, $V_{\rm Switch} = 0$, varying $V_{\rm SD}$.

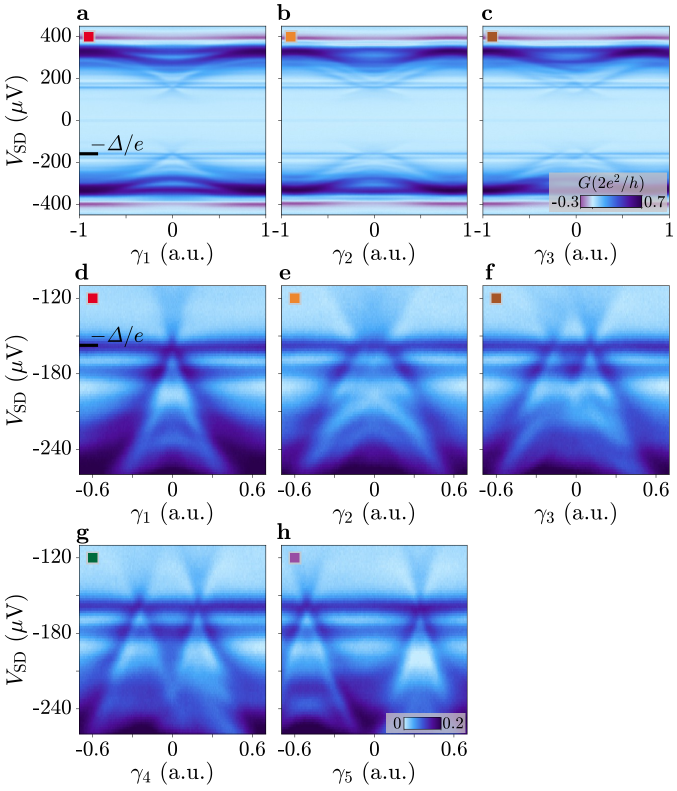

Supplementary Fig. 8. **Tunnelling conductance along phase space linecuts at $V_{\rm G} = -66$ mV. a–c**, Differential conductance G as a function of voltage bias $V_{\rm SD}$ along the linecuts γ_i (coloured arrows in Fig. 7a), for $V_{\rm Switch} = 0$. **d–h**, As **a–c**, but plotted over restricted ranges of $V_{\rm SD}$ and γ_i.

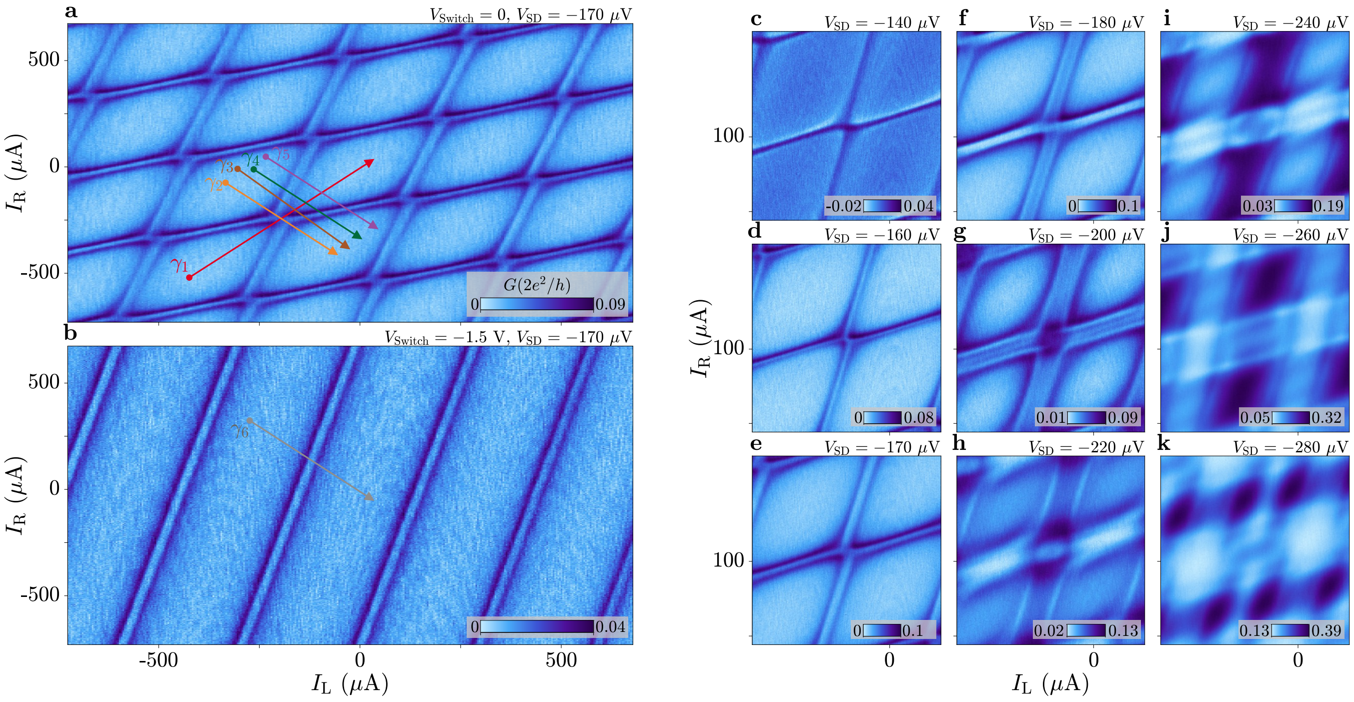

Supplementary Fig. 9. **Constant-bias planes as a function of the two phases at** $V_{\mathrm{G}} = -150$ **mV. a–k**, Differential conductance G as a function of the currents I_{L} and I_{R} injected into the flux-bias lines at fixed values of voltage bias V_{SD}. **a**, $V_{\mathrm{Switch}} = 0$, $V_{\mathrm{SD}} = -170\ \mu$V. **b**, $V_{\mathrm{Switch}} = -1.5$ V, $V_{\mathrm{SD}} = -170\ \mu$V. **c**–**k**, $V_{\mathrm{Switch}} = 0$, varying V_{SD}.

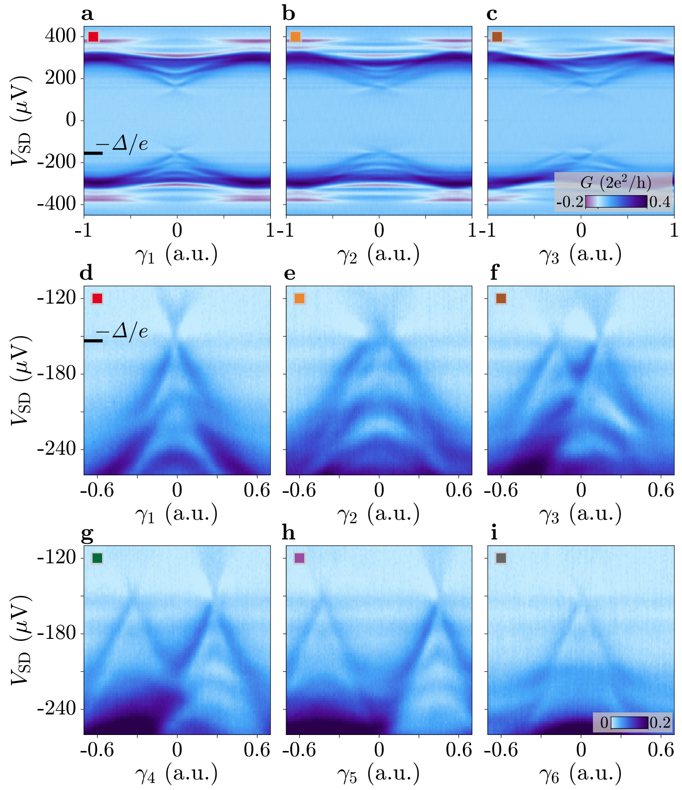

Supplementary Fig. 10. **Tunnelling conductance along phase space linecuts at** $V_{\mathrm{G}} = -150$ **mV. a–c**, Differential conductance G as a function of voltage bias V_{SD} along the linecuts γ_i (coloured arrows in Fig. 9a), for $V_{\mathrm{Switch}} = 0$. **d**–**h**, As **a**–**c**, but plotted over restricted ranges of V_{SD} and γ_i. **i**, As **d**–**h**, but along linecut γ_6 (defined in Fig. 9b), for $V_{\mathrm{Switch}} = -1.5$ V.

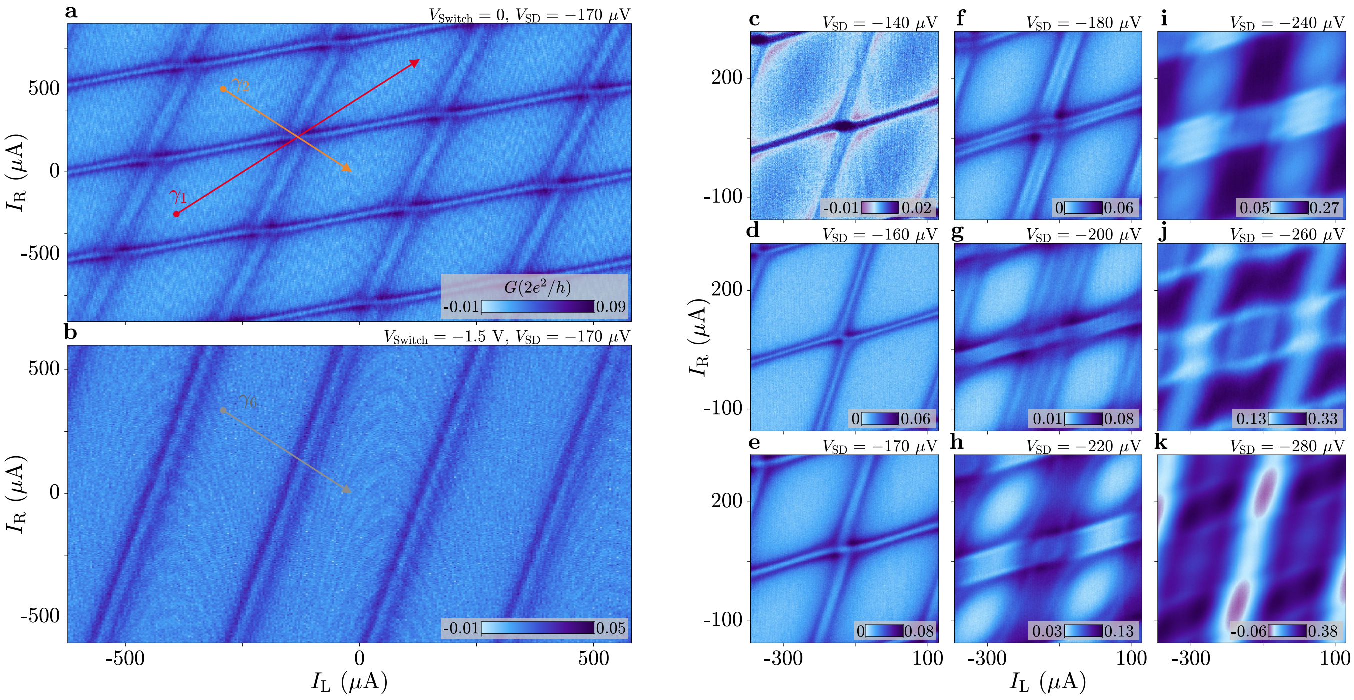

Supplementary Fig. 11. **Constant-bias planes as a function of the two phases at** $V_G = -250$ **mV. a–k**, Differential conductance G as a function of the currents I_L and I_R injected into the flux-bias lines at fixed values of voltage bias V_{SD}. **a**, $V_{Switch} = 0$, $V_{SD} = -170$ μV. **b**, $V_{Switch} = -1.5$ V, $V_{SD} = -170$ μV. **c–k**, $V_{Switch} = 0$, varying V_{SD}.

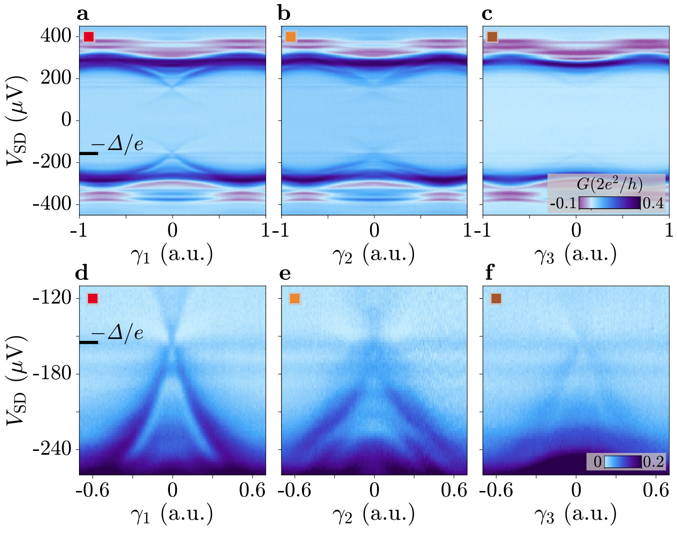

Supplementary Fig. 12. **Tunnelling conductance along phase space linecuts at** $V_G = -250$ **mV. a–c**, Differential conductance G as a function of voltage bias V_{SD} along the linecuts γ_i (coloured arrows in Fig. 11a,b). $V_{Switch} = 0$ in **a**, **b** and $V_{Switch} = -1.5$ V in **c**. **d–f**, As **a–c**, but plotted over restricted ranges of V_{SD} and γ_i.

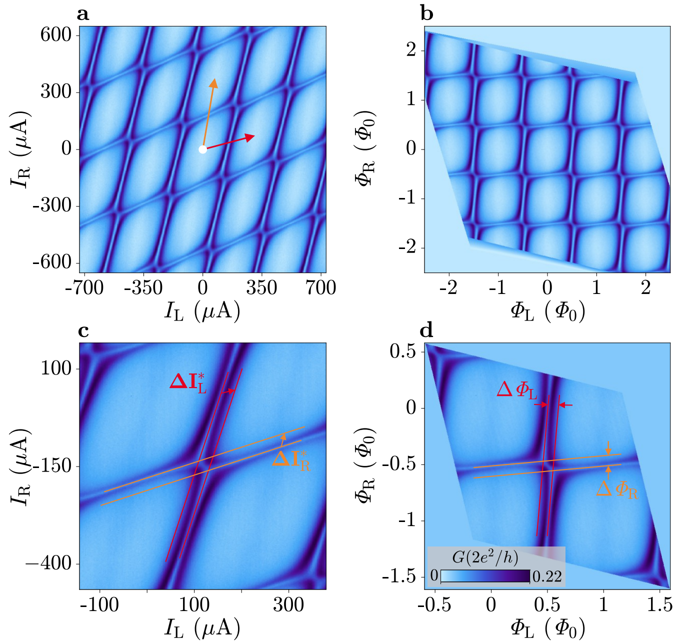

Supplementary Fig. 13. **Current-to-flux remapping and phase shift analysis.** **a**, Differential conductance G as a function of the currents I_L and I_R injected into the flux-bias lines at fixed voltage bias $V_{SD} = -170\ \mu$V (repetition of Fig. 1d from the Main Text). The red and orange arrows indicate the periodicity axes, namely the directions of the external magnetic flux axes Φ_L and Φ_R. The white dot and the end points of the red and orange arrows correspond to the (Φ_L, Φ_R) points $(0, 0)$, $(\Phi_0, 0)$ and $(0, \Phi_0)$, respectively. **b**, As **a**, but remapping the (I_L, I_R) axes to the (Φ_L, Φ_R) axes by using Eqs. 13 and 14 (see text for details). **c**, G as a function of I_L and I_R at $V_{SD} = -165\ \mu$V (repetition of Fig. 4e from the Main Text). The red (orange) lines follow the middle dip between the two resonances of the $\Phi_{L(R)}$-dependent Andreev bound state, on both sides of the intersection point. The $\Phi_{L(R)}$ shift is indicated by the vector $\mathbf{\Delta I}^*_{L(R)}$, whose direction is parallel to the $\Phi_{L(R)}$ axis on the (I_L, I_R) plane (see **a**). **d**, As **c**, but remapping the (I_L, I_R) axes to the (Φ_L, Φ_R) axes. The phase shifts $\Delta\Phi_L$ and $\Delta\Phi_R$, by construction parallel to the axes, are indicated by the arrows.

SUPPLEMENTARY REFERENCES

[1] Pillet, J.-D., Benzoni, V., Griesmar, J., Smirr, J.-L. & Girit, Ç. O. Nonlocal Josephson effect in Andreev molecules. *Nano Lett.* **19**, 7138–7143 (2019).

[2] Kornich, V., Barakov, H. S. & Nazarov, Y. V. Fine energy splitting of overlapping Andreev bound states in multiterminal superconducting nanostructures. *Phys. Rev. Res.* **1**, 033004 (2019).

[3] Kornich, V., Barakov, H. S. & Nazarov, Y. V. Overlapping Andreev states in semiconducting nanowires: Competition of one-dimensional and three-dimensional propagation. *Phys. Rev. B* **101**, 195430 (2020).

[4] Pillet, J.-D., Benzoni, V., Griesmar, J., Smirr, J.-L. & Girit, Ç. Scattering description of Andreev molecules. *SciPost Phys. Core* **2**, 009 (2020).

[5] Beenakker, C. W. J. Random-matrix theory of quantum transport. *Rev. Mod. Phys.* **69**, 731–808 (1997).

[6] Kurilovich, P. D., Kurilovich, V. D., Fatemi, V., Devoret, M. H. & Glazman, L. I. Microwave response of an Andreev bound state. *Phys. Rev. B* **104**, 174517 (2021).

[7] Tinkham, M. *Introduction to Superconductivity* (Dover Publications, 2004), 2 edn.

[8] Pillet, J.-D. *et al.* Andreev bound states in supercurrent-carrying carbon nanotubes revealed. *Nat. Phys.* **6**, 965–969 (2010).

[9] Nichele, F. *et al.* Relating Andreev bound states and supercurrents in hybrid Josephson junctions. *Phys. Rev. Lett.* **124**, 226801 (2020).

[10] Kjaergaard, M. *et al.* Quantized conductance doubling and hard gap in a two-dimensional semiconductor–superconductor heterostructure. *Nat. Commun.* **7**, 12841 (2016).

[11] M_{geom} was calculated analytically as the mutual inductance between two rectangular loops of width $a = 3.5\ \mu$m and height $b = 25\ \mu$m separated along the direction of their width by the distance $c = 0.5\ \mu$m (i.e., half the width of the Al strips constituting the loops in our devices), using $M_{\text{geom}} = \frac{\mu_0 b}{2\pi} \ln \frac{(a+c)^2}{c(2a+c)}$. Here, μ_0 is the vacuum magnetic permeability.

[12] Ter Haar, A. C. J. *Single and coupled Josephson junction quantum bits.* Ph.D. thesis, TU Delft (2005).

[13] Paauw, F. G. *Superconducting flux qubits: Quantum chains and tunable qubits.* Ph.D. thesis, TU Delft (2009).

[14] Annunziata, A. J. *et al.* Tunable superconducting nanoinductors. *Nanotechnology* **21**, 445202 (2010).

[15] van der Ploeg, S. H. W. *et al.* Controllable coupling of superconducting flux qubits. *Phys. Rev. Lett.* **98**, 057004 (2007).

[16] Menke, T. *et al.* Demonstration of tunable three-body interactions between superconducting qubits. *Phys. Rev. Lett.* **129**, 220501 (2022).
