# Supplementary material for: Phase-engineering the Andreev band structure of a three-terminal Josephson junction
Source: Nat Commun. 2023 Oct 25;14:6784. doi: 10.1038/s41467-023-42356-6 (PMC10600130; doi:10.1038/s41467-023-42356-6)
Supplement: Supplementary file 2 — Peer Review [file 41467_2023_42356_MOESM2_ESM.pdf]

nature portfolio

Peer Review File

Phase-engineering the Andreev band structure of a three-terminal Josephson junction

Open Access This file is licensed under a Creative Commons Attribution 4.0 International License, which permits use, sharing, adaptation, distribution and reproduction in any medium or format, as long as you give appropriate credit to the original author(s) and the source, provide a link to the Creative Commons license, and indicate if changes were made. In the cases where the authors are anonymous, such as is the case for the reports of anonymous peer reviewers, author attribution should be to 'Anonymous Referee' followed by a clear attribution to the source work. The images or other third party material in this file are included in the article's Creative Commons license, unless indicated otherwise in a credit line to the material. If material is not included in the article's Creative Commons license and your intended use is not permitted by statutory regulation or exceeds the permitted use, you will need to obtain permission directly from the copyright holder. To view a copy of this license, visit http://creativecommons.org/licenses/by/4.0/.

REVIEWER COMMENTS

Reviewer #1 (Remarks to the Author):

REPORT on “Phase-engineering the Andreev band structure of a three-terminal Josephson junction” that you were recently invited to review for Nature Communications (NCOMMS-23-19114-T) by Fabrizio Nichele

In the manuscript the authors address on interesting topic : the Andreev band structure of a three-terminal Josephson junction. The Andreev bound states (ABS) of two junctions overlap as they share a commun normal region (a 2DEG). This overlap leads to level hybridization and hence changes the energy spectrum of each junction. At the end it simulates an Andreev molecule. This result is not very surprising by itself but it opens up new perspectives in the field of multi-terminal Josephson junctions which has been recently suggested as an interesting platform for topological matter.

The junctions are obtained by coupling two aluminium superconducting electrodes through a In As quantum well.

The ABS energy depends on the phase difference. In this work, phase bias of the two Josephson junctions is provided by independently applying a magnetic flux through two superconducting loops connected to each junction forming the Andreev molecule. One of the two loops is interrupted by a large Josephson junction that can be switched « on « or « off » to probe the ABS spectra when the phase bias of one of the two junctions is « on » or « off ».
Spectroscopy of the ABS in the InAs normal region shared by the two junctions is provided by a tunnel contact.

The experiment is performed with care and the results sound correct. The device is properly designed and a clear hybridization of the ABS as a function of the phase difference of the two junctions is measured by spectroscopy. It is supported by a simple perturbation theory whose results described in more details in the supplementary information are actually quite predictable.

The only criticism of the manuscript is that as hybridization is expected on a simple qualitative analysis a more quantitative matching between theory and experiment would have led to deeper insights on the physics of this device also in view of future works.

As a minor remark, I think that the manuscript can be shorter, for instance, the duality with a double quantum dot addressed in the discussion sounds not very essential. Hybridization is a quite natural phenomenon and so largely encounter in physics that it doesn’t require any specific similarity to be elucidated. A sentence at the end of the main text sounds unclear : » Enhanced spin-orbit-induced effects could be achieved directly in the same material by modifying …on the scale of the spin-orbit length (about 150 nm for InAs).

Reviewer #2 (Remarks to the Author):

In “Phase-engineering the Andreev band structure of a three-terminal Josephson junction” Coraiola et. al report tunneling spectroscopy of a three-terminal (L,M,R) Josephson junction with phase control. One of the loops is interrupted by a second, large Josephson junction. The second junction is used as as witch to effectively tune between a three-terminal and two-terminal configuration. Two Andreev bound states, identified in tunneling spectroscopy, are identified as being predominantly associated with the L-M and M-R junctions. When tuned into resonance, they interact. A model is provided that qualitatively accounts for the authors’ observations. The manuscript makes the concrete claims of the realization of an Andreev molecule with controlled hybridization. There is no doubt that these claims are true. The authors suggest that their data demonstrate the feasibility of engineered Andreev matter in general, and I also agree with this claim.

The presented data are clean and the interpretation is compelling. The success of this work is likely to inspire groups to follow down the path of phase-controlled, coupled Andreev bound states. The “duality” to a capacitively-coupled double quantum dot is also interesting.

I have a few questions and comments below. Once these are answered, I would support publication in Nature Communications.

The “double-dot” picture is quite clear from the data in Fig. 1d, but it would not be clear from the device structure alone. It is also clear from the second device that this situation is reproducible. Still, since it’s not obvious from the design, I ask that the authors provide some more discussion of how this situation is achieved. Are there particular tuneup practices or design criteria that were followed or that the authors believe are important?

The authors state “in this configuration, the probe was coupled very weakly to the 3TJJ and had no influence on the superconducting phases of the rest of the circuit.” It is clear that the probe behaves as a tunnel junction, but to say that it has no influence on the phases is quite a strong statement. I ask the authors to provide more support for this statement (perhaps in the supplement) or alter the language.

The authors state “On either side of the spectrum, we notice two differential conductance peaks with no dependence on γi located at VSD = ±155 µV and ±175 µV.” I do not agree with this statement. These lines appear to interact and even disappear when the Andreev bound state is at a lower energy than the lines.

The authors continue to state that “The first [line] is attributed to the multiple Andreev reflection peak at ±Δ/e, while the second is likely a feature specific to the tunneling probe of this device.” The authors should provide more discussion on this. What is the relationship between multiple Andreev reflections and the observed Andreev molecule? Also, if the second feature is associated with the tunneling probe, why does it depend on the energy of the Andreev states?

The author observe broad features at biases below the gap, which they associate with a broadening of the spectrum of the tunnel probe. This seems plausible. Can the authors roughly estimate how

much of a broadening is required, for instance as a Dynes parameter? Is this parameter consistent with “hard gap” claims in this field, or is it perhaps a more sensitive measure of the gap hardness?

The data in this work is extremely high quality. Since traditional superconducting and semiconducting experiments use quite different filtering strategies, I ask the authors to give details on what filters they used, if light-tight enclosures were used, and so on.

Finally, I would be grateful if the authors could comment on why no spin physics is observed here. The structure dimension is comparable to the spin-orbit length in InAs, and as far as I understand spin-orbit states should be split at certain biases, but this doesn’t appear to be the case. Is this a problem of resolution? Is there some surprise here?

Reviewer #3 (Remarks to the Author):

I have reviewed the manuscript dealing with a three-terminal Josephson junction setup tunable by two independent fluxes and I am quite impressed by both the experimental achievements as well as very convincing theoretical modelling of the experimental findings. The work presents results for one device in the main text strongly supported by a plausible effective model of the junction whose simulation semi-quantitatively agrees with the experimental data plus analogous data for a second device in the supplementary information. Thus, the observed effects as well as their physical interpretation seem robust and reliable. In fact, almost all the question raised during reading the main text were aptly answered in the supplement. There is almost nothing left. Yet, I will pose two minor issues which might be addressed by the authors before the final publication of this high-quality manuscript:

1. I am somewhat confused by the (repeated) comments on the slope difference between the on and off state of the junction (caption of Fig. 1 and end of the first section on p.3). It's not clear to me at the first place why this is so important (it's repeatedly mentioned by I haven't found any more detailed explanation of this phenomenon later). It's somewhat similar with the phase shifts although there is a whole section 6 in the supplement devoted to them. Yet, I would appreciate a better level of presentation of these issues already in the main text, at least pointing specifically to the proper place(s) in the supplementary information from the main text and perhaps also a few more words on the physical origin and significance of these effect already within the mentions in the main text.

2. If I understand correctly the flow of logic of the theoretical modelling, the continuous model description of Ref. [2] in the supplement is only applicable for weak coupling between the ABSs and can be successfully emulated by another model, namely that of quantum dots with discrete levels. The nice correspondence between these two approaches is in fact shown in Figs. S.1 and S.3 for weak coupling. However, for simulating the experiment the tunnel coupling value is almost two orders of magnitude larger than used in the benchmark (t=1.1Delta in the main text vs. t=0.05Delta in Fig. S.3). Despite the very good correspondence with the experimental data, is there any a priori theoretical reason to expect applicability of an effective QD model to the continuous "2DEG" setup with such a strong mutual coupling of the ABSs?

I recommend the manuscript for publication in Nature Communications after these two comments are appropriately addressed (as assessed by the editor).

Response to Reviewer Report of Nature Communications manuscript NCOMMS-23-19114-T "Phase-engineering the Andreev band structure of a three-terminal Josephson junction" by M. Coraiola *et al.*

Reviewer #1

[…] The experiment is performed with care and the results sound correct. The device is properly designed and a clear hybridization of the ABS as a function of the phase difference of the two junctions is measured by spectroscopy. It is supported by a simple perturbation theory whose results described in more details in the supplementary information are actually quite predictable.

We thank the Reviewer for evaluating the manuscript and their positive comments about our work.

The only criticism of the manuscript is that as hybridization is expected on a simple qualitative analysis a more quantitative matching between theory and experiment would have led to deeper insights on the physics of this device also in view of future works.

We agree with the Reviewer that a quantitative matching between theory and experiment might lead to a deeper understanding of the physics of our devices. In the present case, a minimal model was enough to capture the key novelties of our work:

- avoided crossings between Andreev states in Fig. 3c-e, Fig. 3b and Fig. 4a-c;
- band structure anisotropy (Fig. 3c,d);
- low-energy band structure tomography (Fig. 4a-c).

Reproducing all these features, also with semi-quantitative agreement, required only five free parameters (plus a broadening parameter), suggesting that our observations are general and do not rely on specific details.

We acknowledge that we could have commented in more detail on the matching between theory and experiments. Therefore, we have expanded the discussion concerning the model (section "Andreev molecule model and numerical simulations"), further commenting on quantitative agreement (middle of right column). We also better explain the minimal set of parameters required to qualitatively capture the features discussed above (middle of left column).

As a minor remark, I think that the manuscript can be shorter, for instance, the duality with a double quantum dot addressed in the discussion sounds not very essential. Hybridization is a quite natural phenomenon and so largely encounter in physics that it doesn't require any specific similarity to be elucidated.

We acknowledge that the manuscript could be shorter. We have amended it in several parts and, after including the discussion requested by the Reviewers, it is slightly shorter than the initial submission, but with more content. We admit that the quantum dot analogy was very helpful to us, to better understand our own data. Also, Reviewer 2 explicitly mentions that the quantum dot analogy was particularly interesting, therefore we opted for keeping that discussion but rephrased the paragraph (middle paragraph of section "Discussion") to be more concise.

A sentence at the end of the main text sounds unclear : » Enhanced spin-orbit-induced effects could be achieved directly in the same material by modifying …on the scale of the spin-orbit length (about 150 nm for InAs).

We agree that we used a convoluted sentence. We have added a short paragraph at the end of the "Discussion" section to better explain the role of spin-orbit interaction, instead of commenting on it in the outlook. In the present case, we believe that effects related to spin-orbit interaction are suppressed due to the short separation between pairs of terminals in our devices. We referred to a spin-orbit length of ~150 nm, which is longer than the separation between L and R leads in the 3TJJ (100 nm) and substantially longer than the L-M and R-M separations (30 nm). Enlarging the size of our devices is therefore a requirement to resolve spin-orbit-related effects. Indeed, we anticipate that we recently resolved spin splitting of Andreev states in similar devices, but with scattering regions as large as 300 nm x 250 nm (manuscript in preparation).

Reviewer #2

[…] The presented data are clean and the interpretation is compelling. The success of this work is likely to inspire groups to follow down the path of phase-controlled, coupled Andreev bound states. The "duality" to a capacitively-coupled double quantum dot is also interesting. I have a few questions and comments below. Once these are answered, I would support publication in Nature Communications.

We thank the Reviewer for their accurate description of our work, and their nice comments regarding its quality. We address their concerns point-by-point in the discussion below.

The "double-dot" picture is quite clear from the data in Fig. 1d, but it would not be clear from the device structure alone. It is also clear from the second device that this situation is reproducible. Still, since it's not obvious from the design, I ask that the authors provide some more discussion of how this situation is achieved. Are there particular tuneup practices or design criteria that were followed or that the authors believe are important?

We agree that this aspect of our work needs additional explanations. The double-dot picture is a natural consequence of the shape of our device. In particular, the minimal separation between contacts L-M and R-M is very short (30 nm, here the two "single dots" are defined) while the separation between contacts L-R is longer (100 nm). The two "dots" are coupled by their proximity, via a common normal region or superconducting terminal. Please note that, despite the analogy with isolated quantum dots (with sizeable charging energy), our "dots" are strongly coupled (to the leads and among them), as they arise in the highly conductive channels of an open system.

We note that our devices did not need extensive design optimization to achieve the results shown in this work. In addition, tuning was essentially limited to finding a suitable regime to perform tunneling spectroscopy. As shown in the Supplementary Information, results were robust and coherent in different regimes of gate voltage V_{G} and in a second device.

In short, we believe the essential requirements to observe the double-dot picture are:

- Realization of two discrete, separated Andreev states, ideally with near-unity transmission to maximize the visibility of hybridization in the spectrum. This is achieved in our devices by making two short and narrow junctions between terminals L-M and between R-M, where discrete and highly transmissive modes form.

- The two modes must be spatially overlapping. In our geometry, the high-transmission modes are in close proximity to each other.

We have added a sentence in the section "Discussion" (end of second paragraph) to clarify the analogy between a double quantum dot system and our geometry, concisely reflecting the above discussion.

The authors state "in this configuration, the probe was coupled very weakly to the 3TJJ and had no influence on the superconducting phases of the rest of the circuit." It is clear that the probe behaves as a tunnel junction, but to say that it has no influence on the phases is quite a strong statement. I ask the authors to provide more support for this statement (perhaps in the supplement) or alter the language.

We agree that our statement was too categoric. We have therefore used the wording "its influence on the rest of the circuit was limited" in the revised manuscript.

Our reasoning is that the coupling of the probe to the three-terminal Josephson junction (3TJJ) region is so weak (7% in the Main Text, estimated from the high-bias conductance) that any supercurrent between probe and scattering region is suppressed. The full dependence of conductance on V_{T} is shown in Fig. S1a, with the regime under study at $V_{\mathrm{T}} = -1.07\ \mathrm{V}$. On the contrary, the other three leads are strongly coupled to the scattering region. Experiments performed at different transmission, but always in the tunneling regime (see Figs. S9-S12), demonstrate a quantitatively similar spectrum, again supporting the idea that the tunneling probe did not affect the phases between the L, R and M contacts.

The authors state "On either side of the spectrum, we notice two differential conductance peaks with no dependence on γi located at VSD = ±155 µV and ±175 µV." I do not agree with this statement. These lines appear to interact and even disappear when the Andreev bound state is at a lower energy than the lines.

The authors continue to state that "The first [line] is attributed to the multiple Andreev reflection peak at ±Δ/e, while the second is likely a feature specific to the tunneling probe of this device." The authors should provide more discussion on this. What is the relationship between multiple Andreev reflections and the observed Andreev molecule? Also, if the second feature is associated with the tunneling probe, why does it depend on the energy of the Andreev states?

We believe this comment is particularly important and should be discussed thoroughly.

First, we note that the expression "with no dependence on γ_i" that we used in the manuscript was imprecise. The intended meaning was that, unlike the conductance peaks associated with Andreev bound states (ABSs), these peaks are approximately horizontal, namely they do not significantly move in energy as the phase changes. This expression was reformulated in the revised manuscript to "whose position in bias does not appreciably vary with γ_i" (Main Text section "Andreev dispersion along phase space linecuts", middle of left column, and end of Supplementary Note 3).

Second, we feel confident in interpreting the peaks at the gap edge $\pm\Delta/e$ (here ±155 µV) as multiple Andreev reflection (MAR). These peaks are common in Devices 1 and 2, and were also seen in previous works [Nichele et al., Phys. Rev. Lett. **124**, 226801 (2020)].

Conversely, the second pair of peaks (here at ±175 μV) is seen only in Device 1. We did not observe them in Device 2 or in other devices that we studied for different experiments, hence we concluded that they are sample-specific. We associated this second pair of peaks to the superconducting probe, due to the fact that their position in energy does not appreciably change with the phase. Instead, the peaks stay horizontal, which would be unexpected if they were strongly coupled to a superconducting terminal of the 3TJJ. Their origin might be related to mesoscopic defects located in the probe itself or in the region where the tunneling barrier is formed, providing a resonant transport channel. Another possible explanation, which we conceived while writing this response, is that the peaks at ±175 μV originate from a region in the device with a larger superconducting gap, possibly deep under the Al leads. Following the request of the Reviewer to include more discussion, we suggested these potential origins of the peaks at ±175 μV in the revised manuscript (Main Text section "Andreev dispersion along phase space linecuts", near the end of the left column) and further commented at the end of Supplementary Note 3.

So far, we have discussed the potential origin of these conductance peaks but not their dependence on the phase (in intensity or as a small energy modulation) or the possibility of an interaction with the ABSs. In the present case, we believe our data does not support any interaction between horizontal features and Andreev states. The apparent interaction visible in Fig. 2 originates, in our opinion, from the complications of superconductor-insulator-superconductor (SIS) spectroscopy.

We reproduce here Fig. 2a of the Main Text, but plotted with a diverging colorscale that enhanced the contrast and visibility of the horizontal features (Fig. E1). It appears to us, from this picture, that the conductance of the horizontal features is simply added to the conductance of the Andreev states and of the background. We mark four regions where the background conductance is particularly low (yellow and green contours), which we believe are characteristic features of SIS spectroscopy (often even negative differential conductance features are observed). We observe that the modulation of the peak at ±155 μV, that seems to bend near the crossing with the ABS around γ_1=0, is correlated to the presence of two low-conductance lobes in proximity of the gap edge (green contours), that locally reduce the conductance of the peak. Similarly, the peak at ±175 μV has a lower conductance for γ_1 closer to ± 1, where the background conductance is lower (yellow contours) and is enhanced when it crosses the Andreev state (orange contours). In conclusion, in this simple explanation, conductance modulations of the peaks at ±155, ±175 μV are related to a variation in the background conductance, without the need to invoke a direct interaction with the ABSs or their energy.

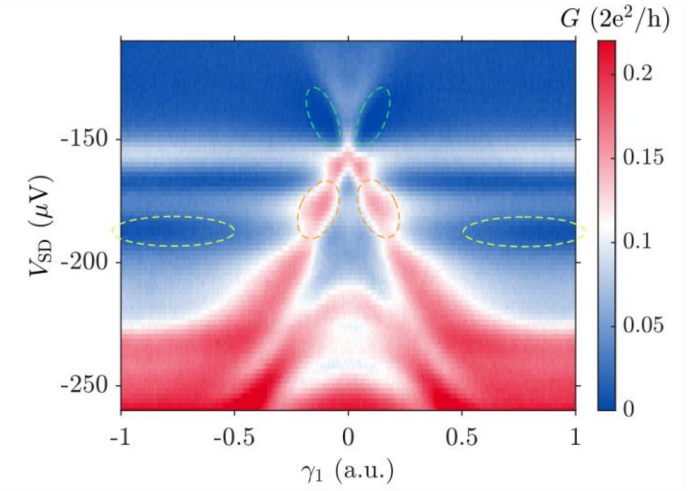

FIG. E1. Differential tunneling conductance G as a function of bias voltage V_{SD} along phase space linecut γ_1 (zoom-in of Fig. 2a from the Main Text). Dashed colored contours highlight the regions of varied background conductance described in the text.

Most importantly, we do not observe that the dispersion of Andreev states changes appreciably when crossing these horizontal peaks, suggesting the absence of an interaction between them. In fact,

besides the modulation of the peaks, we might expect a distortion of the *shape* of the ABSs, which instead seems to simply cross these resonances (with a conductance enhancement). This is more apparent for the dataset of Fig. S10 of the Supplementary Information, where $V_{\mathrm{G}} = -150$ mV and the probe transmission was reduced by approximately a factor 2 compared to the data of the Main Text. Here, the peaks at ±155, ±175 µV are very feeble and the interplay with the ABSs is significantly weaker, but the shape of the ABSs is similar. Finally, in Device 2 there is no second set of resonances and the shape of the ABSs is similar to that of Device 1. This suggests that the presence of the peaks at ±175 µV in Device 1 is not crucial for the qualitative trend of its ABSs.

To reflect these arguments, we have added a sentence in the revised Main Text (Main Text section "Andreev dispersion along phase space linecuts", end of the left column), stating that the modulation of the peaks at ±155, ±175 µV with γ_i might be due to the variation of the background conductance. We note that the shape of the ABSs is not distorted as they cross these peaks. We also provided additional comments in the Supplementary Information for the description of the datasets at $V_{\mathrm{G}} = -150$ mV (Note 4, near the end of the second bullet point) and of Device 2 (end of Note 3), as discussed above.

The author observe broad features at biases below the gap, which they associate with a broadening of the spectrum of the tunnel probe. This seems plausible. Can the authors roughly estimate how much of a broadening is required, for instance as a Dynes parameter? Is this parameter consistent with "hard gap" claims in this field, or is it perhaps a more sensitive measure of the gap hardness?

First, we note that, to study directly the hardness of the gap, superconductor-insulator-superconductor (SIS) tunneling spectroscopy is not ideal, since the resulting differential conductance as a function of the voltage bias requires a convolution between two relatively complex densities of states. A direct study would require the use of a normal probe, i.e., NIS spectroscopy [Chang et al., Nat. Nanotechnol. **10**, 232-236 (2015); Kjaergaard et al., Nat. Commun. **7**, 12841 (2016)]. For this reason, we do not believe that our devices constitute a more sensitive measure of the gap hardness than established techniques. In our case, the SIS spectroscopy is further complicated by the presence of discrete subgap states, thus the exact spectral form of the density of states is not known a priori. Nevertheless, the differential conductance near zero voltage bias is always approximately zero in our measurements, and the smearing is comparable to previous reports which performed spectroscopy with a normal probe and supported the claim of a hard gap [Kjaergaard et al., Nat. Commun. 7, 12841 (2016)].

The closest situation to SIS spectroscopy (without subgap states) is achieved when both superconducting phases are near zero, as in Fig. 2a of the Main Text near one edge of the γ_i range. In Fig. E2 (below), the blue trace shows a linecut of the dataset of Fig. 2a at $\gamma_1 = 0.95$. The two highest conductance peaks (red arrows) appear when two BCS peaks are aligned and display a finite broadening, as expected. We also note additional conductance peaks in the inner slopes of such peaks (green arrows) that are not compatible with pure SIS spectroscopy; from Fig. 2a, these peaks are related to ABS resonances that do not reach the gap edge. The orange dashed line shows the result of the convolution [Pillet et al., Nat. Phys. **6**, 965-969 (2010); Nichele et al., Phys. Rev. Lett. **124**, 226801 (2020)] between two BCS densities of states with Dynes parameter $\delta = 0.04$, where a Lorentzian-broadened peak was added to one of the two (see Appendix below for further details). We observe that the combination between a Dynes parameter and additional broadened subgap states qualitatively reproduce the broadening in this configuration. A Dynes parameter of a few percent, that we believe to be a reasonable estimate, is very similar to previous reports [Pillet et al., Nat. Phys. **6**,

965-969 (2010); Nichele et al., Phys. Rev. Lett. **124**, 226801 (2020)], but alone it is not sufficient to reproduce the observed broadening, since the high-energy subgap states also play a role.

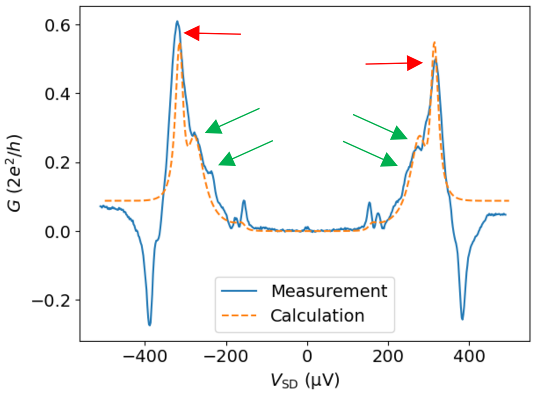

FIG. E2. Differential conductance G as a function of voltage bias V_{SD}. Blue line: linecut of Fig. 2a of the Main Text at $\gamma_1 = 0.95$. Orange dashed line: calculation of the differential conductance based on two BCS-like densities of states, both with Dynes parameter $\delta = 0.04$ and with a Lorentzian-broadened state included in one of the two. See Appendix for further details on the calculation. Colored arrows highlight the features described in the text.

We believe that the main content of this discussion should be included in the manuscript. In the revised version, we expanded the comment on the broadening with an additional sentence, suggesting the potential origin of the broadening (section "Andreev dispersion along phase space linecuts, middle of left column). We also added two sentences in Supplementary Note 2 (end of first paragraph).

The data in this work is extremely high quality. Since traditional superconducting and semiconducting experiments use quite different filtering strategies, I ask the authors to give details on what filters they used, if light-tight enclosures were used, and so on.

We thank the Reviewer for their appreciation regarding the data quality. Surprisingly, our experiments were performed with relatively simple filtering and no light-tight enclosures. We have added a description of the measurement setup in the section "Methods" of the revised manuscript at the beginning of "Measurement techniques".

Finally, I would be grateful if the authors could comment on why no spin physics is observed here. The structure dimension is comparable to the spin-orbit length in InAs, and as far as I understand spin-orbit states should be split at certain biases, but this doesn't appear to be the case. Is this a problem of resolution? Is there some surprise here?

Spin physics is of great interest in the field of hybrid multiterminal Josephson junctions. In the present case, we see at least two reasons why no spin splitting of Andreev states is observed.

The separation between L-M and R-M terminals is very short compared to the spin-orbit length in InAs, hence no spin splitting of Andreev states forming in those junctions is expected. The separation between L and R terminals (100 nm) is also shorter than the spin-orbit length. When the tunneling probe is tuned, the electron density in the scattering region is likely decreased, resulting in further increase of the spin-orbit length. Finally, we do not observe a direct high-transmission channel between L and R. In this scenario, we expect no spin splitting to arise due to the physics of multiterminal Josephson junctions. For instance, the seminal paper on spin-splitting in three-terminal junctions [van Heck et al., Phys. Rev. B **90**, 155450 (2014)] considered a junction length larger than the spin-orbit length by a factor 10 and direct coupling of all three terminals to the scattering region.

Spin-splitting is also expected for long junctions with strong spin-orbit interaction [Tosi et al., Phys. Rev. X **9**, 011010 (2019)]. In the present case, the superconducting coherence length in InAs is about 600 nm ($\xi_{\mathrm{InAs}} = \sqrt{\hbar v_{\mathrm{F}} l_e / \pi \Delta}$ with v_{F}: Fermi velocity; l_e: electron mean free path; Δ: induced superconducting gap), which makes our junctions much closer to the short-junction limit. We therefore expect a spin splitting much smaller than that measured by Tosi et al. (1 GHz or 4 ueV), making it impossible to resolve with DC spectroscopy.

We have added a paragraph at the end of the section "Discussion" of the revised manuscript to give some insights regarding the absence of spin physics, instead of addressing the point very briefly at the end of the Main Text.

Reviewer #3

I have reviewed the manuscript dealing with a three-terminal Josephson junction setup tunable by two independent fluxes and I am quite impressed by both the experimental achievements as well as very convincing theoretical modelling of the experimental findings. The work presents results for one device in the main text strongly supported by a plausible effective model of the junction whose simulation semi-quantitatively agrees with the experimental data plus analogous data for a second device in the supplementary information. Thus, the observed effects as well as their physical interpretation seem robust and reliable. In fact, almost all the question raised during reading the main text were aptly answered in the supplement. There is almost nothing left. Yet, I will pose two minor issues which might be addressed by the authors before the final publication of this high-quality manuscript:

We are grateful to the Reviewer for evaluating the manuscript and for their very positive feedback. We address the two issues in the following.

1. I am somewhat confused by the (repeated) comments on the slope difference between the on and off state of the junction (caption of Fig. 1 and end of the first section on p.3). It's not clear to me at the first place why this is so important (it's repeatedly mentioned by I haven't found any more detailed explanation of this phenomenon later). It's somewhat similar with the phase shifts although there is a whole section 6 in the supplement devoted to them. Yet, I would appreciate a better level of presentation of these issues already in the main text, at least pointing specifically to the proper place(s) in the supplementary information from the main text and perhaps also a few more words on the physical origin and significance of these effect already within the mentions in the main text.

We agree that our initial formulation could cause confusion. The change of slope is probably not the most interesting of our observations. However, as we present the first measurements of this kind, we wanted to discuss all our observations in detail. We also agree that it is stylistically wrong to discuss the same topic multiple times in the Main Text.
In short, we think that the change of slope of Andreev states between Fig1d and Fig1e is an experimental indication that the loops are coupled. It is consistent and likely has the same origin of the phase shifts. Quantitative analysis (described in Supplementary Note 6) further points to a Josephson-like mutual inductive coupling, analogous to what was discussed in two works in the context of superconducting qubits [van der Ploeg et al., Phys. Rev. Lett. **98**, 057004 (2007); Menke et al., Phys. Rev. Lett. **129**, 220501 (2022)]. Clearly, this coupling is only present when the switch junction is transmissive, as otherwise no current could circulate in the right loop.
Following the advice of the Reviewer, we grouped the discussions on the slope change at the end of Supplementary Note 6, to which we refer in the Main Text (end of section "Andreev molecule and

numerical simulations”). Since the physical origin of the phase shift and the slope difference is the same (based on the above discussion), we have extended the comment on the phase shift at the end of the section “Andreev molecule model and numerical simulations” to the slope difference. We emphasized its physical origin and added the two references on the topic [van der Ploeg et al., Phys. Rev. Lett. **98**, 057004 (2007); Menke et al., Phys. Rev. Lett. **129**, 220501 (2022)], previously only provided in the Supplementary Information.

2. If I understand correctly the flow of logic of the theoretical modelling, the continuous model description of Ref. [2] in the supplement is only applicable for weak coupling between the ABSs and can be successfully emulated by another model, namely that of quantum dots with discrete levels. The nice correspondence between these two approaches is in fact shown in Figs. S.1 and S.3 for weak coupling. However, for simulating the experiment the tunnel coupling value is almost two orders of magnitude larger than used in the benchmark (t=1.1Delta in the main text vs. t=0.05Delta in Fig. S.3). Despite the very good correspondence with the experimental data, is there any a priori theoretical reason to expect applicability of an effective QD model to the continuous "2DEG" setup with such a strong mutual coupling of the ABSs?

We acknowledge that the discussion on the quantum dot model deserves more explanation. Despite being referred to as “quantum dot (QD) model”, this should not be mistaken as a model that describes the physics of isolated QDs weakly coupled to the leads. Conversely, the two QDs are very strongly coupled to the superconducting leads (i.e., the Γ parameters are large with respect to the superconducting gap Δ), which suppresses the energy dependence and makes the model suited to study open systems, such as a continuous 2DEG. The QD model hence provides a very flexible tool to describe generic scattering regions and is well adapted to the present case. In fact, Andreev bound states in Josephson junctions are fully described by their transmission [Kornich et al., Phys. Rev. Res. **1**, 033004 (2019)] and, for describing their spectrum, any sensible microscopic model incorporating such parameter may be chosen. Our QD model further enables numerical solutions for large coupling strength where the analytical approach cannot be followed. As was shown, starting with a QD model one can reproduce results for semiconductor-based junctions even in an open regime [Galzman et al., PRB **104**, 174517 (2021)]. Finally, the fact that single channels are included in the QD model does not constitute a limitation, since the experiments show that only a few channels are involved in the formation of the Andreev molecule.

We have expanded the discussion concerning the QD model in Supplementary Note 1 (end of first page) to include this content.

I recommend the manuscript for publication in Nature Communications after these two comments are appropriately addressed (as assessed by the editor).

Appendix (for Response to Reviewer #2): calculation of differential conductance from convolution of densities of states

Here we describe the calculation that was used to simulate the differential conductance $G(V_{\mathrm{SD}})$ in Fig. E2 (orange trace). We consider two energy-dependent density of states profiles $\rho_1(E)$ and $\rho_2(E)$, given by:

$$\rho_1(E) = \mathrm{Re}\left\{\frac{|E|}{\sqrt{(E + i\delta\Delta)^2 - \Delta^2}}\right\} \quad (1)$$

$$\rho_2(E) = \rho_1(E) + A \cdot \left[\frac{w}{(E - E_0)^2 + w^2} + \frac{w}{(E + E_0)^2 + w^2}\right] \quad (2)$$

where $\delta = 0.04$ is the Dynes parameter [Dynes et al., Phys. Rev. Lett. **41**, 1509–1512 (1978)] and $\Delta = 157\ \mu\mathrm{eV}$ the superconducting gap. The second term in Eq. 2 represents a Lorentzian-broadened subgap state centered at energy $\pm E_0 = \pm 127\ \mu\mathrm{eV}$ and with parameters $w = 25\ \mu\mathrm{eV}$ and $A = 35\ \mu\mathrm{eV}$. In Appendix Figure (below) we show $\rho_{1,2}(E)$ (normalized).

The differential conductance G as a function of voltage bias V_{SD} is obtained as [Pillet et al., Nat. Phys. **6**, 965-969 (2010); Nichele et al., Phys. Rev. Lett. **124**, 226801 (2020)]:

$$G(V_{\mathrm{SD}}) \propto \frac{\partial}{\partial V_{\mathrm{SD}}} \int_E [f(E - eV_{\mathrm{SD}}, T) - f(E, T)]\rho_1(E - eV_{\mathrm{SD}})\rho_2(E)\mathrm{d}E$$

where $f(E, T)$ is the Fermi-Dirac distribution function and $T = 30\ \mathrm{mK}$ the temperature. This expression leads to the $G(V_{\mathrm{SD}})$ trace shown in Fig. E2, up to a scaling factor for the vertical axis.

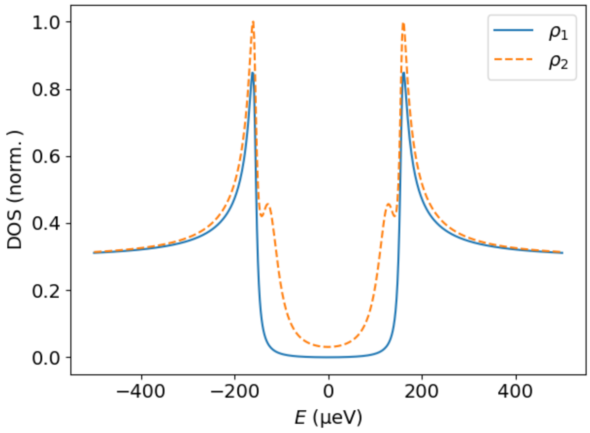

Appendix Figure: normalized densities of states ρ_1 and ρ_2 as a function of energy E, described by Equations (1) and (2) respectively.

REVIEWER COMMENTS

Reviewer #1 (Remarks to the Author):

In the revised manuscript, the authors add some discussion to elucidate the agreement between theory and experiment. For the sake of clarity and transparency, I believe that this should be push further.

The ABS spectrum expected for the model in the inset of Fig.3a is shown in Fig.3a. The authors focus on the experimental features captured by this model. It would be interesting to see also if and where the model fails.

In the response to the referee comments the authors says :

« a minimal model was enough to capture the key novelties of our work:
• avoided crossings between Andreev states in Fig. 3c-e, Fig. 3b and Fig. 4a-c;
• band structure anisotropy (Fig. 3c,d);
low-energy band structure tomography (Fig. 4a-c). »

Now, I'm afraid to say that point 1 is trivial. Point 2 is more interesting. This anisotropy seems also to be visible in the energy of the ABS. Indeed Fig.3e clearly shows that the maximum energy of the two ABS is different. In Fig. 3a this asymmetry persists away from the avoided crossing point at phi_R=phi_L=pi. However in the data in Fig. 2h, the maximum energy of the two ABS looks the same (-155 ueV). Therefore it seems that there is here a qualitative discrepancy between the model and the data. Point 3 is a bit confusing as Fig. 4a-c focus in a very low energy scale (smaller than 0.2*Delta) while the data go up to about Delta (Fig. 4l). Fig.4 is beautiful but it is very difficult to see whether or not model and data agree approaching the gap energy scale. Why not presenting data and theory for the same energy range and discuss also possible discrepancy ? As a minor remark, to help in reading this figure it would be useful to express the energy in Fig. 4a-c not in the gap unity but in ueV as done in Fig. 4d-l.

In one word, as an avoided crossing of ABS seems a natural phenomenon and the theory remains at the level of a qualitative description of the experimental results (for instance no coupling parameter, t, is obtained by fitting the data), the manuscript would be improved by honestly saying which features of the model are not observed and why. Missing explanations are also allowed.

Reviewer #2 (Remarks to the Author):

The authors have responded to all of my questions, in particular about features at +/- 155 uV, +/- 175 uV, Dynes parameter, lack of spin physics, and my request for details in their filtering. I found their responses and the changes to the manuscript to be very helpful and instructive. From my perspective, this manuscript is definitely ready to be published.

Reviewer #3 (Remarks to the Author):

I have carefully read the authors' replies to all referees' comments and reviewed the updated manuscript. I am satisfied with the reactions to my questions and as much as I can judge also with the replies to other referees' comments. Therefore, I recommend the updated manuscript for publication in Nature Communications.

Response to Reviewer Report of Nature Communications manuscript NCOMMS-23-19114-T "Phase-engineering the Andreev band structure of a three-terminal Josephson junction" by M. Coraiola *et al.*

Reviewer #1

In the revised manuscript, the authors add some discussion to elucidate the agreement between theory and experiment. For the sake of clarity and transparency, I believe that this should be push further.

We thank the Reviewer for their further feedback on the revised manuscript.

The ABS spectrum expected for the model in the inset of Fig.3a is shown in Fig.3a. The authors focus on the experimental features captured by this model. It would be interesting to see also if and where the model fails. In the response to the referee comments the authors say: « a minimal model was enough to capture the key novelties of our work:

- avoided crossings between Andreev states in Fig. 3c-e, Fig. 3b and Fig. 4a-c;
- band structure anisotropy (Fig. 3c,d);
- low-energy band structure tomography (Fig. 4a-c). »

Now, I'm afraid to say that point 1 is trivial. Point 2 is more interesting. This anisotropy seems also to be visible in the energy of the ABS. Indeed Fig.3e clearly shows that the maximum energy of the two ABS is different. In Fig. 3a this asymmetry persists away from the avoided crossing point at phi_R=phi_L=pi. However in the data in Fig. 2h, the maximum energy of the two ABS looks the same (-155 ueV). Therefore it seems that there is here a qualitative discrepancy between the model and the data.

The asymmetry in Fig. 3e is not linked to the band anisotropy but is a consequence of the different transmission coefficients of the conduction channels that give rise to the two ABSs. This is accounted for by our model with the choice of transmission parameters (0.998 for the left ABS, 0.992 for the right ABS), as described in Supplementary Note 1. In fact, there is no reason why the two states should have identical transmission in a real device. This asymmetry is present in the data, despite it might be hard to spot from Fig. 2f–h due to the finite experimental resolution. In the figure below we try to highlight it with markers and guide to the eyes. In the simulations, the difference between the maximum energies is approximately $0.023\Delta \approx 3.5\ \mu eV$, consistent with Fig. 2. The different transmission probability between left and right state is observed much better in Fig. 4d,e as different width in the ABSs contours.

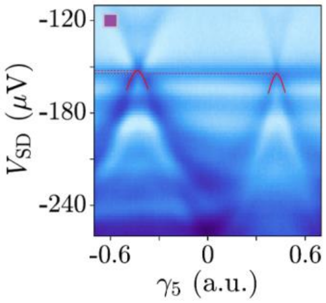

Fig. E1: Replica of Fig. 2h of the Main Text. Red annotations mark the maximum energies of the two Andreev bound states, highlighting the slight difference between them. The different heights originate from different transmissions of the two states and are not a result of hybridization.

In conclusion, the difference in the energy maxima in Fig. 2h is not a result of hybridization but is a property of individual ABSs which is taken into account in our model. To better clarify this important

point, we now comment on the different transmission probabilities in the Main Text, reporting the two transmission parameters (section "Andreev molecule model and numerical simulations", middle of left column).

Point 3 is a bit confusing as Fig. 4a-c focus in a very low energy scale (smaller than 0.2*Delta) while the data go up to about Delta (Fig. 4l). Fig.4 is beautiful but it is very difficult to see whether or not model and data agree approaching the gap energy scale. Why not presenting data and theory for the same energy range and discuss also possible discrepancy?

We apologize for creating confusion on this point. We choose a model that accounts exclusively for the two most transmissive ABSs. Data and simulations are compared at low energy because there the two states hybridize, without other states present. At energies closer to the gap, comparing data and simulation is pointless because additional states present in the device are purposely not included in the model.

Based on the simulated spectra shown in Fig. 3a and 3c-e, no particularly interesting features appear in the higher energy range when only two modes are present. In Fig. E2 below we replot Fig. 4g of the Main Text (right), where an additional ABS appears, and compare it to a simulated cut plane at the corresponding energy, $E = -0.29\Delta$ (left). This simulation qualitatively reproduces the features associated with the two high-transmission ABSs in Fig. 4g, but it does not add insights compared to Fig. 4c and, naturally, does not capture the additional modes (see red arrows). We therefore believe that simulations at higher energies do not add relevant information to the manuscript but could instead be misleading for the readers.

The reason for comparing simulations and experiment only at low energy was already discussed in the manuscript towards the end of the section "Tomography of the Andreev band structure". We have now rephrased and expanded it to further improve the clarity of the manuscript.

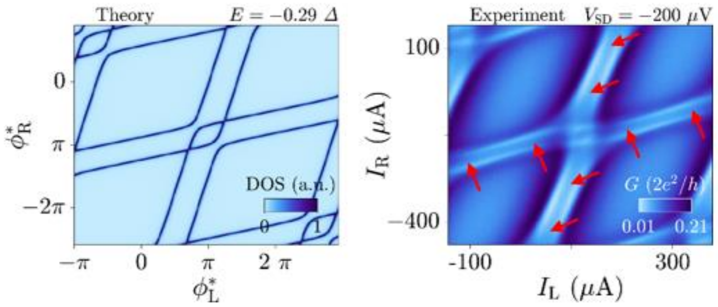

Fig. E2: (Left) As in Fig. 4a of the Main Text, for energy E=-0.29 Δ. (Right) Replica of Fig. 4g of the Main Text. Red arrows indicate the first low-transmission Andreev states appearing in the spectrum.

As a minor remark, to help in reading this figure it would be useful to express the energy in Fig. 4a-c not in the gap unity but in ueV as done in Fig. 4d-l.

We agree with the reviewer that, in general, a direct comparison of the two labels might help reading the figure. However, in the present case, it is important to express the energy in Fig. 4a-c in gap units:

- In the experimental maps, the applied bias is shifted with respect to the energy by an amount $\Delta/e \approx 155$ μV (due to the superconducting probe). Converting the labels in Fig. 4a-c to energy would result to $-9, -14, -26$ μeV respectively, making the comparison to the voltage bias labels $-150, -165, -180$ μV misleading.

- The model does not assume a certain value of the gap Δ, instead all quantities are expressed in units of Δ. Changing the units in Fig. 4a-c (and consequently in Fig. 3) to energy by assuming a certain value of Δ would be an unnecessary loss of generality, while the simulations are currently valid for any Δ and hence relevant for various material platforms.

In one word, as an avoided crossing of ABS seems a natural phenomenon and the theory remains at the level of a qualitative description of the experimental results (for instance no coupling parameter, t, is obtained by fitting the data), the manuscript would be improved by honestly saying which features of the model are not observed and why. Missing explanations are also allowed.

We sincerely thank the Referee for these comments. We hope the revised manuscript better addresses the comparison between theory and experiments. In the new manuscript, we better explain what choices were made in the definition of the model and what agreement is expected and observed between simulations and data. In summary:

- The model does not account for states close to the gap edge, as those modes are purposely not included in the simulations. This is now stressed better in the manuscript (section "Tomography of the Andreev band structure", near the end).
- The different heights of the ABSs simulated in Fig. 3a are a consequence of the transmission coefficients associated to the two ABSs alone, and they were chosen based on the experimental data. We feel there is very good agreement between data and simulations on this point. The transmission parameters are now explicitly written in the text (section "Andreev molecule model and numerical simulations", middle of left column).
- The avoided crossing is not reproduced exactly, but the agreement is well within an order of magnitude. This is expressed in the section "Andreev molecule model and numerical simulations" (middle of right column): "all simulations qualitatively reproduce the key features observed in the measurements […] and lead to the same order of magnitude for the avoided crossings".
- The model does not capture the large phase shifts present in the experiment. This discrepancy is stated and explained at the end of the section "Andreev molecule model and numerical simulations". We also discuss this point in greater detail in Supplementary Note 6.

REVIEWERS' COMMENTS

Reviewer #1 (Remarks to the Author):

The authors elucidate the links between experiment and theory.

I still don't see the interest on presenting Figures 4 g-l as these data are not discussed in the text and as clearly explained in the response to the referee at energy close to the gap additional states appear that are not included in the model. I think that these 6 panels (g to l) of Figure 4 can be move in the Supplementary Information.

Otherwise I recommend publication

Response to Reviewer Report of Nature Communications manuscript NCOMMS-23-19114-T "Phase-engineering the Andreev band structure of a three-terminal Josephson junction" by M. Coraiola *et al.*

Reviewer #1

The authors elucidate the links between experiment and theory.

I still don't see the interest on presenting Figures 4 g-l as these data are not discussed in the text and as clearly explained in the response to the referee at energy close to the gap additional states appear that are not included in the model. I think that these 6 panels (g to l) of Figure 4 can be move in the Supplementary Information.

Otherwise I recommend publication.

We thank the Reviewer for their comment. We fully agree that it is not appropriate to present the data of Fig. 4g-l without a proper discussion in the text. Therefore, we have expanded the section "Tomography of the Andreev band structure" (p. 4, bottom of right column), commenting on the most important features of Fig. 4g-l.

We believe that it is important that the full tomography of the two-dimensional band structure is presented in the Main Text, despite some data fall outside the validity range of the theory model. This is the first measurement of its kind and will constitute a baseline for future studies in more advanced devices.
